# Effects of Pyrolysis Control Parameters on the Structural Properties of Biomass‐Derived Activated Carbon Materials and Their Energy Applications

**DOI:** 10.1002/tcr.202500268

**Published:** 2025-12-08

**Authors:** Meenal Gupta, Maria F. Gaele, Pasquale Gargiulo, Yogesh Kumar, Valeria Califano, Aniello Costantini, Tonia M. Di Palma

**Affiliations:** ^1^ CNR‐Istituto di Scienze e Tecnologie per la Mobilità Sostenibile Naples Italy; ^2^ Dipartimento di Ingegneria Chimica dei Materiali e della Produzione Industriale Università degli Studi di Napoli Federico II Naples Italy; ^3^ Department of Physics ARSD College University of Delhi New Delhi India

**Keywords:** activated carbons, energy conversion and storage devices, gas diffusion layer, hardwood, hardwood‐activated carbons, pyrolysis, softwood, softwood‐activated carbons

## Abstract

The development of sustainable and low‐cost energy storage and conversion systems is crucial for modern society. To enable large‐scale implementation, research has focused on synthesizing eco‐friendly and cost‐effective components, particularly electrolytes and electrodes, for electrochemical devices such as fuel cells, supercapacitors, and batteries. Carbon‐based materials are widely employed as electrodes or catalyst supports, and biomass‐derived carbons have emerged as attractive alternatives due to their abundance, renewability, and low cost. The physicochemical and electrochemical properties of biomass‐derived activated carbons (ACs) including porosity, surface area, and electrical conductivity strongly depend on their synthesis and activation processes. This review analyzes the preparation of ACs from various biomass sources, emphasizing pyrolysis in tubular furnaces and the influence of parameters such as activation temperature, time, gas flow rate, and carbonization conditions. The relationships between these parameters and the resulting structural and electrochemical properties are discussed, with a particular focus on plant‐derived carbons. Finally, the applications of biomass‐derived ACs as electrode materials in different electrochemical systems are summarized, highlighting how precursor type and synthesis route govern their performance and suitability for sustainable energy technologies.

## Graphical abstract

Tubular pyrolysis synthesis is a widespread method to produce activated carbons (ACs). This review focuses on the different controlling parameters of the tubular pyrolysis synthesis method and their influence on the performance of synthesized ACs, thereby considering both experimental aspects and the use of plant‐based biomasses (PBBs) as feedstock. Strategies to tune the morphology and electrical properties of ACs using softwood, hardwood, and other waste PBBs are discussed. This study paves the way for the effective use of such materials as electrodes for energy applications.

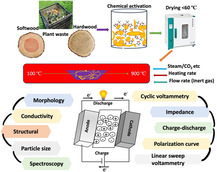

## 1 Introduction

To protect the environment from CO_2_ emissions and other pollution issues, wood resource‐based activated carbons (ACs) should be produced from the perspective of the circular economy. Indeed, the annual production of woody products may increase to 3.1 billion m^3^ by 2050, resulting in a large amount of available raw materials [1].

Wood‐derived activated carbons (WDACs) have excellent mechanical, thermal, mass transport, ionic (due to attached functional groups), optical, fluidic, and electronic properties [2, 3]; therefore, they have great potential for environmental issues such as water pollution [4] and energy scarcity. Moreover, ACs based on biomass precursors, such as lignocellulosic biomass, micro‐ and macroalgae feedstock, and other biomasses grafted with functional groups, such as the carboxyl group, or modified with surface hydrophobicity and decorated with metal nanoparticles have shown potential for use in various other applications [5, 6, 7, 8, 9]. Algae, lignocellulosic, and waste biomasses are major precursors for producing activated carbons (ACs). Algae biomass grows rapidly on non‐arable land using wastewater, making it an accessible carbon source, produces moderate to high carbon yield [10]. Lignocellulosic biomass derived from agriculture and forestry offers high carbon yield due to its cellulose, hemicellulose, and lignin content, but requires optimized pretreatment to separate components and facilitate efficient carbon conversion [11]. In particular, lignin‐rich precursors enhance sp^2^‐hybridized carbon formation, improving electronic conductivity [12]. Waste biomasses from municipal and industrial streams are abundant and low‐cost [13], but require sorting, decontamination, and often purification.

Biomass‐derived carbons are valuable as electrode materials and catalysts in energy storage and conversion devices. Heteroatom‐doped and porous ACs improve oxygen reduction reaction/oxygen evolution reaction (ORR/OER) performance while reducing materials cost. Chemical/thermal treatments and nanoparticle incorporation enable the production of functionalized carbons [14, 15, 16] and carbon nanotubes (CNTs) [17], graphene [18, 19], and carbon quantum dots [20, 21], contributing to sustainable waste valorization and reduced fossil fuel dependence. ACs also show strong performance in adsorption and catalytic applications [22, 23, 24, 25, 26, 27, 28, 29, 30, 31, 32, 33]. They have been used for gold recovery [34], biomolecule detection [35], catalytic transformations [36], antibacterial activity [37], and demonstrating their versatility and relevance in energy and environmental technologies [38].

The carbons produced during the pyrolysis can be divided into two categories: crystalline and defective polycrystalline [39, 40]. Graphite and diamond have crystalline structures; however, other carbons are mostly considered polycrystalline, as they have appropriate crystallite sizes with defects and impurities [22, 41]. ACs are anisotropic porous structured materials with high surface areas of up to 2000 m^2^ g^−1^ [42]. ACs are generally reported in powder, fibrous, or granular form [43]. The high surface area of the ACs is attributed to their porous nature. In general, the inner and outer surfaces of ACs are enriched with oxygenated functional groups. Powder‐activated carbons (PACs) have higher surface areas than granular‐activated carbons (GACs) because they have smaller particles. Different particle sizes of PACs (~0.2 mm diameter) and GACs (~5 mm diameter) can be applied for different purposes; however, they are used mainly as adsorbents [44, 45, 46]. Porous and fibrous ACs are more useful for applications involving adsorption from water because of their high aspect ratio, as the length of the fibers is much greater than their diameter [47, 48].

Most of the ACs derived from fossil fuel products, which are used for commercial purposes in a wide range of industrial applications, are expensive to produce [49]. Indeed, the low yield during carbonization explains its high cost, while the combustion of fossil fuels produces greenhouse gases, which make this process unsustainable. Therefore, efforts have been made to synthesize carbons using renewable and biodegradable sources such as biomass sources [24, 29], industrial wastes [50], and municipal waste [51, 52], to make the process more sustainable and cost‐effective [49].

The properties of ACs, such as their carbon content and percentage of other elements present, depend mainly on the precursor used and affect the electrochemical properties of the devices. The minerals present in all kinds of biomasses can function as active sites, which are required for catalytic reactions [53]. The higher carbon content, which is ultimately linked to the electrode conductivity and porosity, can be found in ACs synthesized from wood (trunks, branches, rootstocks, and leaves) [42, 54]. Studies have been reported during the past decade on improved synthesis methods for ACs using woody precursors and the physicochemical properties of the derived ACs; however, identifying a specific plant or part of the plant for the production of ACs with targeted properties for a particular kind of application is still challenging. The researchers reported different physicochemical properties for the produced ACs using same biomass specie (e.g., coconut shell) [4, 55, 56, 57, 58, 59] due to varying synthesis conditions. However, it is also well known that the same precursors, such as moso bamboo (*Phyllostachys pubescens*) and oak barrel wood, grown in different geographical regions may have distinct properties [60, 61] that also affect the final properties of the ACs.

The development towards energy storage devices, for example, supercapacitors, or electrochemical energy conversion and storage devices, for example, batteries and fuel cells, is one of the major needs of modern society, and biomass‐derived ACs (BDACs) have been reported as viable electrode materials. Nevertheless, the use of BDACs as electrode materials for commercial applications is still hampered by the previously mentioned issues. In fact, although ACs exhibit suitable thermal and mechanical properties and favorable physicochemical stability, which allows them to be used at high temperatures, even with various types of electrolytes, but they present variabilities in porosity, surface area, and electronic conductivity, which limit their use at the commercial level. These properties, as well as other morphological, electrical, and mechanical properties, of the produced ACs depend on the chosen synthesis method and the activation or doping materials used. ACs prepared under the same experimental conditions may have varying properties because of the use of different grain sizes and the geographical origin of the precursors used [54, 62, 63].

This review aims to address the challenges faced and the impact of pyrolysis parameters on the properties of developed plant‐derived AC electrode materials, with a main focus on woody precursors, so that it can help researchers to develop more suitable AC materials for different applications. In particular, this review is intended to (i) collect and compare the physical properties of different kind of ACs derived from plant‐based biomasses (PBBs); (ii) study the effects of various controlling parameters of the pyrolysis method and their effects on the resulting ACs; (iii) discuss the utilization of softwood, hardwood and other PBBs in the synthesis of ACs with respect to their properties; and (iv) finally, discuss the use of plant‐based BDACs in various energy devices.

## 2 Wood and its Categories

**FIGURE 1:**
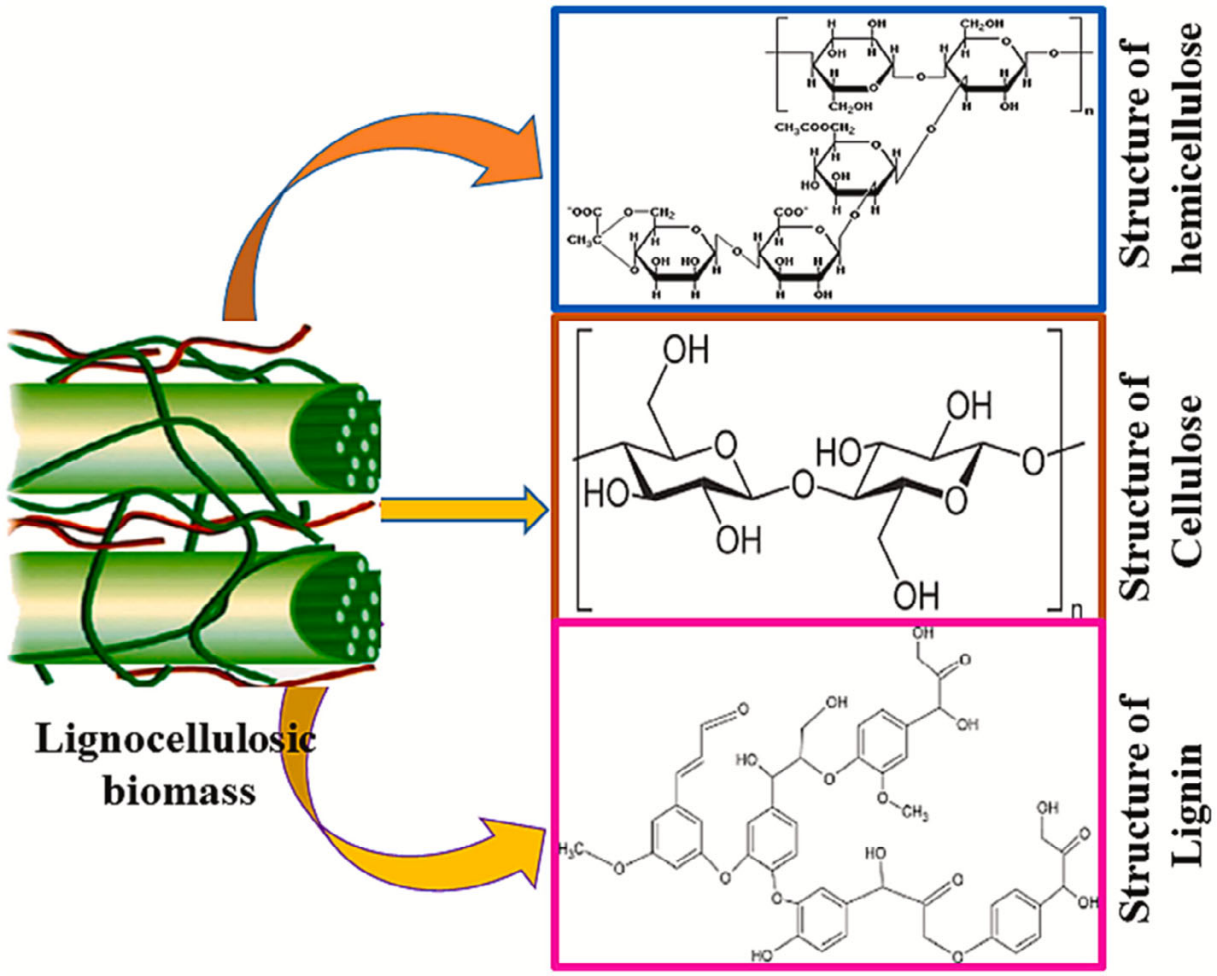
Molecular structure of lignocellulosic biomass components. Reproduced from ref. [66]. Copyright (2024), The Authors. Published by Elsevier.

Wood, considered a renewable solid biomass, is a hierarchical porous material with an anisotropic structure [64]. Wood structures vary as a function of the volume fraction and typology of its cells. Prosenchyma and parenchyma are the main cells of wood. They have different shapes, and their relative ratio varies for different wood types [65]. This ratio defines the conducting, supporting, and storing nature of the cells for a particular wood. The main constitutional components of wood cell walls are cellulose, hemicellulose, and lignin polymers, whose molecular structures are shown in Figure 1 [66]. Non‐structural low‐molecular‐weight organic compounds, such as lipids, terpenoids, resin and fatty acids, waxes, and phenolic compounds, are also present in wood in small amounts [67].

The basic structure of cellulose consists of hydroglucose molecules linked together by β–(1,4)‐glycosidic bonds to form repeated cellobiose units arranged in long linear chains with a high degree of polymerization, which varies from 8000 to 10 000 [68, 69]. Cellulose fibers are mostly crystalline and contain small amorphous regions. Fibers are oriented parallel to the wall cell axis at a certain angle because of the formation of intra/intermolecular hydrogen bonds between the cellulose chains and OH groups [68, 70, 71]. Cellulose has a more crystalline structure than hemicellulose and lignin, which have essentially amorphous structures [72] and barely soluble in most common solvents.

Pentose sugar chains, which comprise mainly xylose, constitute the basic structure of hemicellulose [73]. Xylose units are linked by β–(1,4)‐glycosidic bonds and have branches because of α‐(1,2) glycosidic bonds with 4‐O‐methylglucuronic acid groups [74]. Hemicellulose is easily hydrolyzed with alkalis [74, 75].

The complex lignin structure is formed by alkyl phenols (aromatic compounds) that are chemically bonded with phenylpropane units, which provide structural strength and protect the polymer from degradation [76]. The macrostructure of the lignin complex is highly polar because it has many functional groups, such as hydroxyl, methoxyl, and carbonyl groups [77]. Lignin is a natural aromatic phenolic polymer present in softwoods, hardwoods and other agricultural residues, such as cork or plant substances [63, 78, 79]; accordingly, it can be classified into three categories: softwood, hardwood, and grass lignin.

Lignin was produced in large amounts on an early basis, and it has been widely applied as a fuel. It is also employed for adhesion purposes and in tanning. Owing to the excessive quantities produced every year and its high carbon content, lignin is considered a suitable precursor for preparing ACs [42, 50, 80, 81, 82, 83, 84, 85, 86].

**FIGURE 2:**
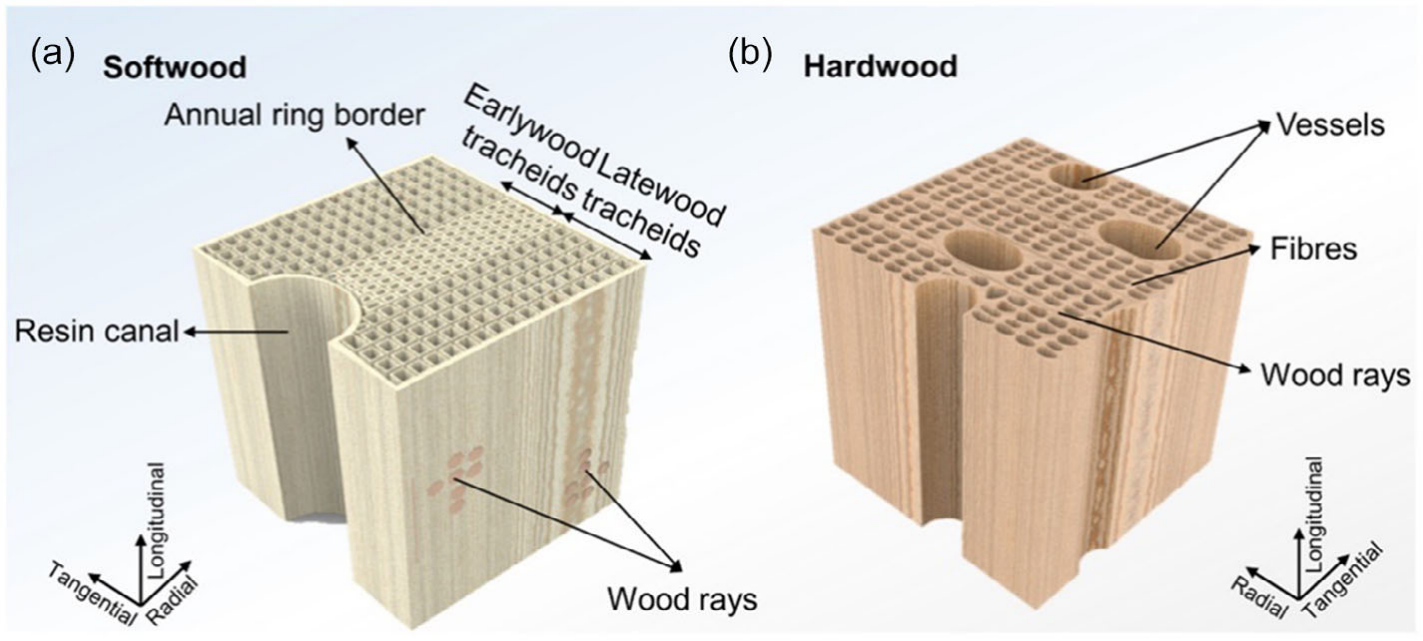
Molecular structure of (a) softwood and (b) hardwood. Reproduced from ref. [87]. Copyright (2024), Elsevier B.V.

On the basis of density, wood can be of two types, namely, hardwood and softwood, with the first exhibiting a higher density than the latter. The molecular structures of softwood and hardwood are shown in Figure 2. Softwood and hardwood both contain oriented cellulose nanofibers that provide strength to the wood and are responsible for the transport of water. The difference between hardwood and softwood at the macroscopic level is presented well in detail by Ruffinatto et al. [88]. NMR spectroscopy, vibrational spectroscopy, X‐ray diffraction, and thermogravimetric analysis have been applied to different woods to classify them into hardwood and softwood [88, 89, 90].

Lignin, cellulose, and hemicellulose have been used in many applications, such as the preparation of ACs, as separated components and together with other lignocellulosic polymers in the form of whole wood. Among them, lignin is particularly suitable for the production of ACs because of its moderate stiffness, high carbon content, and low cost.

### 2.1 Softwood

As mentioned above, prosenchyma and parenchyma are the cell constituents of all woods. In softwoods, 90% of the wood volume is made of tracheids, which are composed mainly of prosenchyma cells [91], and the remaining 10% are parenchyma cells. The function of prosenchyma cells is to provide strength, whereas parenchyma cells have a storage function and help with the transport of fluid cells [65, 86]. Gradually, the cell diameter of tracheid cells decreases, and the cell wall becomes thicker, providing mechanical strength to the wood. The intercellular space, which is present mainly in softwoods, is known as the resin canals. In softwood, lignin is covalently bound to galactoglucomannan [92]. For example, cedar (*Cedrela angustifolia*), douglas fir (*Pseudotsuga menziesii*), juniper (*Juniperus communis* L.), yew (*Taxus baccata*), hoop pine (*Araucaria columnaris*), spruce (*Picea abies* (Karst) L.), larches, redwood (*Sequoia sempervirens*), pine (*Pinus sylvestris* L.), and Chinese fir (*Cunninghamia lanceolata*) woods are softwoods. The lignin contained in softwoods is derived from the assembly of coniferyl alcohol units; thus, it is also referred to as coniferyl or guaiacyl lignin [42]. The molecular structure of softwood lignin is common in different species [42, 93]. Softwood is itself divided into two categories: earlywood and latewood. Earlywood has relatively large cell diameters with a small cell wall tracheid thickness, whereas latewood has a small cell diameter and thick cell wall tracheids [48, 94].

### 2.2 Hardwood

The development of hardwood is an evolutionary process in which the structure of cellulose becomes more complex than that of softwood [65, 91, 95, 96]. Hardwood lignin (dicotyledonous angiosperm lignin) consists of two units, namely, coniferyl and sinapyl alcohol [42], and comprises three types of cells, namely, fibrous tracheids with honeycomb structures to provide structural support, vessel elements with large lumen diameters that aid in water transport, and parenchyma cells [86]. Moreover, compared with softwood, hardwood contains more pits and has a more hierarchical porous structure [65]. Compared with softwood tracheids, vessels decrease in number with time and form channels that transport water more effectively. Compared with softwood vessels, hardwood vessels are larger in diameter, and these vessels are interconnected by holes created at the cell end. In hardwoods, lignin is covalently bonded with xylans. Some examples of hardwood include oak (*Quercus*), teak (*Tectona grandis*), sapele (*Entandrophragma cylindricum*), iroko (*Milicia excelsa*), meranti (*Shorea*), mahogany (*Swietenia macrophylla*), tamarind (*Tamarindus indica*), birch (*Betula pendula*), walnut (*Juglans regia*), ash (*Fraxinus*), beech (*Fagus sylvatica*), poplar (*Populus alba* L.), rosewood (*Dalbergia*), basswood (*Tilia americana*), balsa (*Ochroma pyramidale*), maple (*Acer*), jarrah (*Eucalyptus marginata*), eucalyptus (*Eucalyptus teriticornis*), olive (*Olea europaea*), and coconut wood (*Cocos nucifera*).

## 3 Pyrolysis Method and its Controlling Parameters

Pyrolysis can be used to synthesize ACs starting from different biomasses and woody precursors, such as hardwoods and softwoods. Most pyrolysis methods are facile and eco‐friendly; however, notable attention should be given to the selection of their controlling parameters, as they greatly affect the properties of ACs. In the typical synthesis process, ACs are synthesized using a thermochemical treatment in an inert atmosphere, which involves a series of reactions that depend on the following intermediate steps: pretreatment, functionalization, activation, and doping.

**FIGURE 3:**
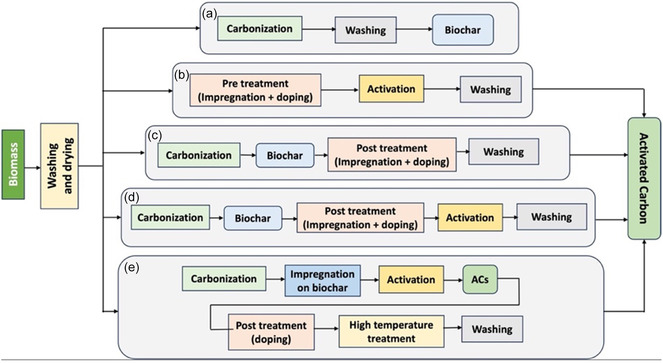
Schematic of the preparation of (a) biochar [97, 98], (b) ACs [99], (c) ACs [100], (d) ACs [101], and (e) ACs [102] using the pyrolysis method.

During the pyrolysis of an untreated biomass, biochar is the main product, with bio‐oil and biogas as byproducts. However, additional steps, such as pretreatment and impregnation, should be included in the preparation process of ACs to obtain improved/controlled porous structures and larger surface areas. Such treatments can be directly applied to either biomass or biochar to modify their physical and chemical properties. Consequently, ACs typically perform better than biochars do, especially when they are employed in energy devices. The most common steps involved in the synthesis of ACs are (i) washing the biomass, (ii) drying, (iii) impregnation, (iv) functionalization/doping, (v) carbonization, and (vi) washing the final product to neutralize its pH [22]. In general, the various methods that can be used to synthesize ACs from a precursor are reported in Figure 3. The selection of the most suitable preparation method for ACs with applications in electrochemical devices depends mainly on the precursor, the activation agent, and other experimental conditions (Figure 3a–e). Single pyrolysis (Figure 3a–c) is cost‐effective and requires less time to prepare biochar/ACs. Double pyrolysis, as shown in Figure 3d,e, increases the cost of the process as well as the preparation time, but it can increase the performance of the obtained ACs in terms of the pore volume and surface area [103].

In general, during the first carbonization step of any biomass precursor (without impregnation), even at low temperatures (300°C – 400°C) (Figure 3a,c–e), different types of gases and tars are released, creating a porous structure [104]. Later, the activation process can be performed on such porous structured biochar using an impregnating material, for example, KOH, which acts as an etching and activating agent [30]. The crystals of the impregnating agent can coat the biochar surface and the inner surface of the pores. During the second carbonization step, as shown in Figure 3c–e, the space preoccupied by the impregnating agent can be transformed into macropores. During carbonization, high temperatures and the amount of impregnated salt have individual effects on the physical properties of the final ACs. At high temperatures, the reaction of the impregnating agent with carbon may lead to the release of different gases, which help in the formation of new micro‐ and mesopores; however, the presence of more impregnating agent helps to increase the surface area by creating larger pores even at lower temperatures [105].

The porous structure of ACs and, therefore, their surface area can also be increased by adopting a double activation method in which precarbonization is applied directly either on the raw precursor or on the impregnated biochar, followed by postactivation treatment [103, 106, 107]. For example, in the study reported by Sajjadi et al. [106] pistachio wood was subjected to preactivation using ammonium nitrate (NH_4_NO_3_) as an activator followed by carbonization. Afterwards, the carbonized product was subjected to another impregnation using NaOH as a second activator to obtain better performance towards Pb(II) sorption from water media, with the obtained surface area reaching 190 m^2^ g^−1^. However, other researchers report ACs prepared using two‐stage pyrolysis with low pore volume and lower surface area than those prepared using single pyrolysis [108]. Therefore, suitable methods for controlling the ratio of micropores and mesopores, and customizing the structure or other properties of ACs during pyrolysis are still under investigation.

The different parameters controlling the pyrolysis process and their effects on the properties of the produced ACs are discussed in the following sections.

### 3.1 Pretreatment of Biomass

The pretreatment process involves either prewashing or soaking of the biomass in various chemicals, such as ethanol [109], NaOH [110], ionic liquids [111, 112], alkali, and alkaline earth metal salts [113], which may lead to the production of more thermally stable ACs and can increase their surface area. In the case of woody precursors, the char yield may decrease because of the impact of such pretreatments [114]. This yield reduction can be justified by the removal of lignin and hemicellulose from the biomass after soaking in the various chemicals. However, the remaining cellulose structure may either remain the same or become deformed, depending on the solvent used and other treatment conditions. Pretreatment helps to produce more crystalline ACs if the cellulose structure is not affected greatly. Additionally, the removal of lignin and hemicellulose helps to create pores or to convert micropores into mesopores inside the structure. For instance, an aqueous mixture of organic solvents (ethanol and acetone) may dissolve up to 60% of the lignin component to produce cellulose‐rich rice straw pulp depending on other pretreatment conditions, such as temperature, residence time, and ethanol concentration [109, 115]. The solubility of hemicellulose is reported to be similar for methanol and ethanol [116]. Xylose and glucose solubility are reported mainly for neutral alcohols; however, alkaline ethanol is responsible for better dissolution of xylose than glucose [116, 117]. Such a soaking process may improve the physicochemical properties of prepared ACs. Menya et al. [114] reported the effect of NaOH soaking on the thermal and chemical properties of rice husk for the preparation of ACs. ACs prepared using soaked rice husk biomass presented better thermal properties with reduced char yield. This improvement is explained mainly by the removal of ash, lignin, and hemicellulose during soaking in NaOH.

Ionic liquids (ILs), for example, 1‐ethyl‐3‐methyl‐imidazolium acetate ([EMIM] OAc) and 1‐butyl‐3‐methyl‐imidazolium chloride ([BMIM]Cl), are used to pretreat rice straw and have been shown to be good sources for removing lignin; however, these compounds also reduce cellulose crystallinity [118, 119, 120, 121]. In addition, ionic liquids can also destroy the organized structure of rice straw by making the surface of the cellulose rough and creating a more porous structure [122, 123]. Nevertheless, ILs are not environmentally friendly; therefore, deep eutectic solvents (DESs), which are greener and cost‐effective, have also been evaluated in biomass soaking, but few such methods have been reported [124, 125, 126]. In contrast to ILs, soaking with a choline chloride (ChCl)‐based DES helps to maintain or even improve the crystallinity of cellulose via the interaction of the hydroxyl groups of DES with the hydrogen bonds of cellulose, resulting in a stable cellulose [124, 125]. DES pretreatment of rice husk biomass has improved the crystallinity of cellulose as well as its porosity by up to a factor of 6 [126]. Both rice husk and wood have similar building components, that is, lignin, cellulose, and lignocellulose; therefore, similar effects may also be observed for woody precursors.

### 3.2 Activation Process

**FIGURE 4:**
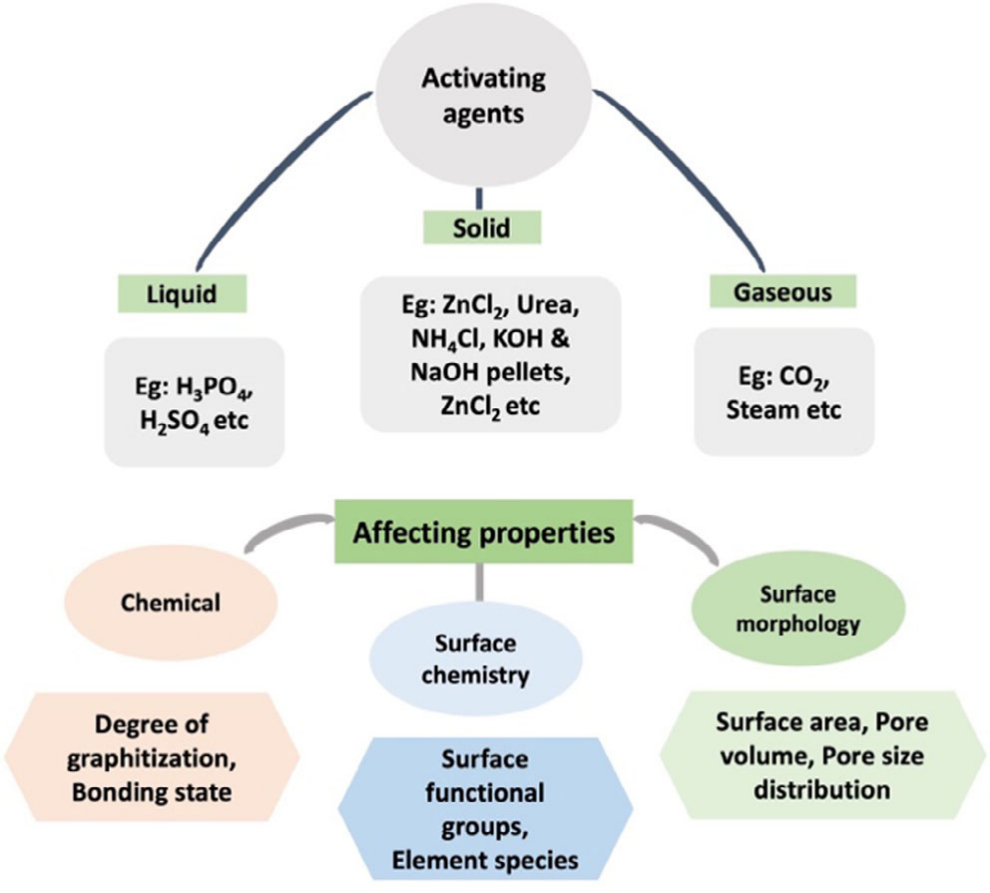
Activating agents and their effects on the resulting carbons.

The activation process is used to reorganize the structure of wood and other PBBs waste precursors by creating pores and other defects [30]. Two types of activation methods have been reported for preparing ACs, namely, chemical activation and physical activation. The main purpose of such a step is to impregnate the precursor to create more regular micropores and structural defects, especially if specific activating agents for the doping with heteroatoms are introduced in the preparation procedure [30, 127]. Compared with physical activation, chemical activation is generally preferred because it is more efficient, requires less time, and can be applied at lower temperatures [105, 128, 129, 130]. However, physical activation has been demonstrated to be more effective if followed by chemical activation [131, 132]. Overall, the various physicochemical properties of the produced ACs, which can be affected by activation processes, are shown in Figure 4.

#### 3.2.1 Chemical Activation

Chemical activation is adopted to impregnate the precursor using various chemical agents, such as H_3_BO_3_, phosphoric acid (H_3_PO_4_), FeCl_3_, H_2_SO_4_, KOH, NaOH, NaCl, Na_2_SO_4_, CaCl_2_, Ca(OH)_2_, HNO_3_, MnCl_2_, zinc chloride (ZnCl_2_), etc. [133, 134, 135, 136], and is followed by carbonization at higher temperatures. KOH and H_3_PO_4_ are considered the most commonly used alkaline and acidic activating agents, respectively. During chemical activation, other substances are extracted, mainly because of the dissolution of lignin, which favors the opening of pores. Chemical activation can be applied at different stages of the preparation procedure: (i) before carbonization, (ii) during the first carbonization, (iii) after the first carbonization, and (iv) before and after the first carbonization. The effects of impregnation/activation at different stages of the procedure on the morphological and structural properties of prepared ACs can vary, and to our knowledge, studies of the effects at these stages are scarce. However, chemical activation at any stage certainly helps to create porosity inside the carbon structure, as the creation of pores due to the dissolution of lignin in rice husk, which was later chemically impregnated using H_3_PO_4_, was reported by Kennedy et al. [136]. In general, acid activation is more effective for lignin and hemicellulose than for cellulose because the amorphous structure of lignin is more susceptible to these treatments than the crystalline structure of cellulose [93].

**FIGURE 5:**
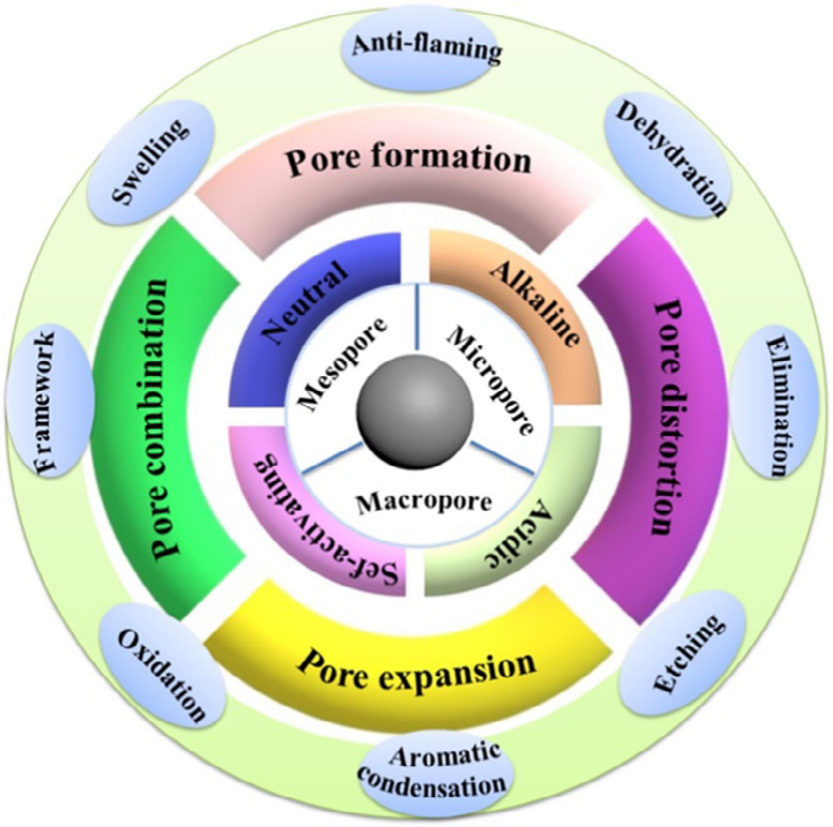
Illustrations of porosity, surface properties, and processes adopted during activation in the presence of different activating driving forces (agents). Reproduced from ref. [137]. Copyright (2020), Elsevier B.V.

Gao et al. [137] summarized in detail the activation mechanism for different types of activating agents responsible for the formation of various types of pores and their effects on experimental methods. Both alkali and acidic treatments have their own peculiar drawbacks, which makes it important to choose activators that are preferable for a particular use and application. The use of acidic activators can corrode metal equipment, whereas hydroxide treatment requires many water washes of the final product to remove the high amounts of alkali metal salts [104]. In general, the activation mechanism for all types of activating agents is shown in Figure 5. The pore formation is linked with the type of activating agent. The pore distribution and pore dynamics are linked phenomenon which affect their possible applications and performance (Figure 5).

During chemical activation, the amount of the activating agent, the impregnation method, and the total impregnation time affect the properties of the final product. These factors are mentioned in the following section.

##### 3.2.1.1 Chemical Activating Agent

**FIGURE 6:**
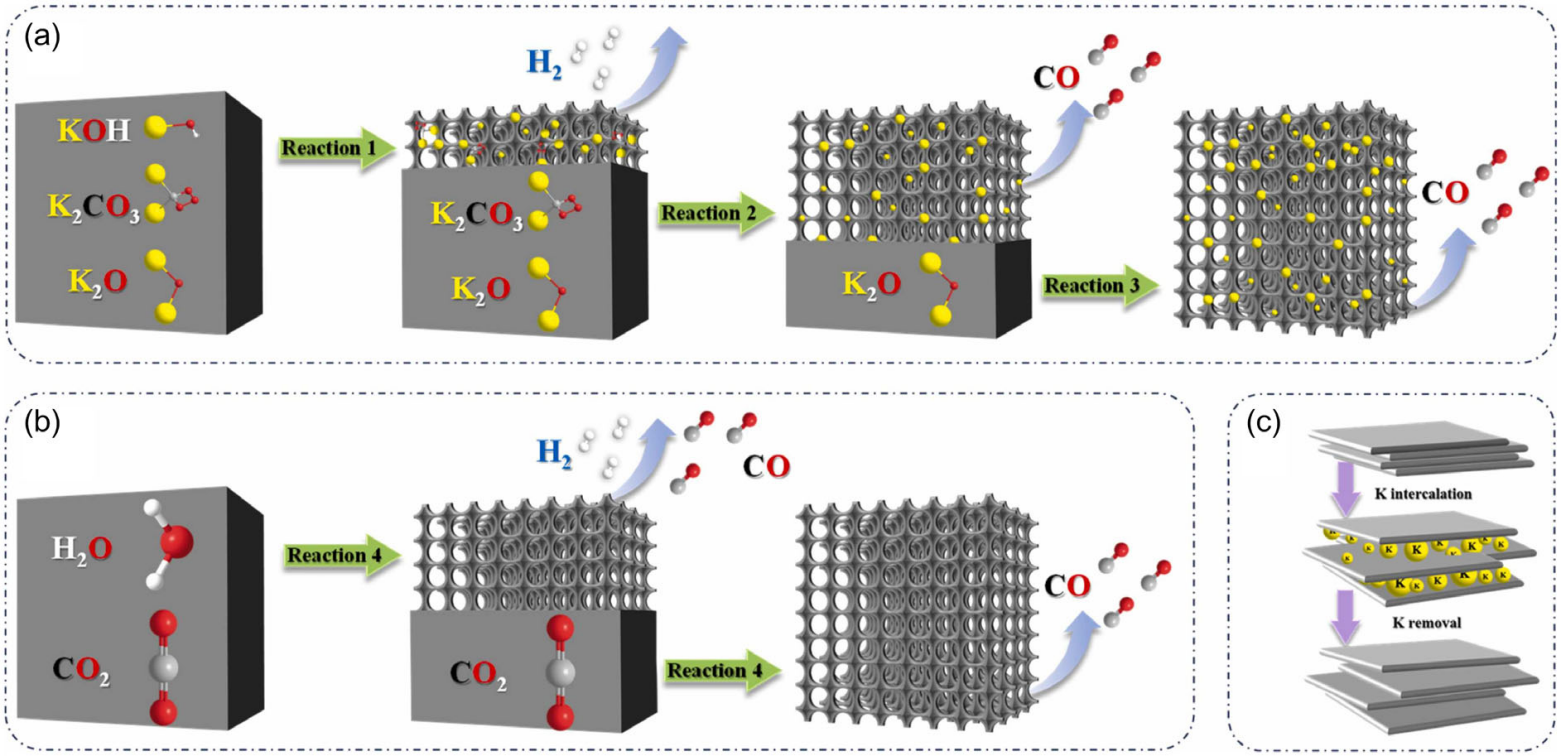
Schematic illustration of the activating processes: (a) KOH working as an activating agent, (b) water and CO_2_ physically activating the resulting carbon, and (c) potassium exfoliating the carbon layers. Reproduced from ref. [102]. Copyright (2024), Elsevier B.V.

As mentioned earlier, among different alkaline and acidic activating agents, KOH, NaOH, ZnCl_2_, K_2_CO_3_, and H_3_PO_4_ are the most reported activating agents. In particular, KOH is currently the most commonly used highly alkaline activation agent [23, 24, 46, 47]. Indeed, KOH helps to develop micropores and enhances the number of ‐OH functional groups on the AC surface, which are known to enhance the wettability of prepared ACs [138]. During the activation process, K_2_CO_3_ and K_2_O are reduced to metallic K by carbon, leading to CO_2_ emission, which creates micropores [102, 139]. The detailed pore creation mechanism using KOH activation was reported by Yang et al. [89, 102] in detail and is shown here in Figure 6.

Generally, the weight ratio of the biomass precursor with respect to the activating agent varies from 1:1 to 1:5 [101, 102, 140, 141, 142], but there are a few reports where this ratio is increased to 1:10 [143], where the activating agent is CuCl_2_ with camphor tree grains as biomass. Single‐step KOH activation on different biomasses (holm oak, coconut shell, and cocoa pod husk) has been reported by various research groups, confirming the main formation of micropores in prepared ACs [144, 145, 146]. The effects of one‐step and two‐step KOH activation on two different biomasses, kanlow switchgrass (*Panicum virgatum* L.) and *Miscanthus*, are presented in detail by Oginni et al. [140]. The one‐step AC exhibited a greater Brunauer–Emmett–Teller (BET) surface area (1596.52 m^2^ g^−1^) for *Miscanthus* BDAC than the 1271.66 m^2^ g^−1^ observed for AC derived from kanlow switchgrass biochar. Qiu et al. [141] studied the effect of the impregnation ratio of KOH on corn straw (1:4 to 1:6) and reported that the use of more KOH during impregnation (i.e., 1:5 rather than 1:4) can induce the collapse of micropores and the development of mesopores, which increase the surface area and mesopore volume; however, after a certain ratio (i.e., for 1:6), the excessive amount of KOH during activation can collapse the pore structure, which may result in a decrease in the surface area by increasing the pore volume [102]. Higher amounts of KOH (beyond 1:4) can decrease the yield of the product, but it is more effective to produce highly meso‐ and microporous‐structured ACs [147, 148, 149] up to a certain ratio of KOH activation. Liu et al. [150] reported that KOH activation is easier on hardwood‐alkali lignin than on softwood‐alkali lignin, as confirmed by FTIR and morphological studies.

After KOH, another highly explored alkaline activating agent is NaOH. During activation, potassium and sodium hydroxide have similar interactions with organic precursors [151, 152]. There are a few reports in which NaOH has been reported to be a better activating agent than KOH because it results in superior adsorption capacity and a more uniform pore distribution of *Eucalyptus*‐based AC [153]. However, a KOH activator may help to increase nitrogen sorption on woody precursors when compared with NaOH [104].

H_3_PO_4_ has also been evaluated as an activating agent for different woody precursors [93, 154, 155, 156, 157, 158]. The activation process with H_3_PO_4_ on lignocellulosic materials can be explained in two steps [93]. First, bond cleavage reactions occur, followed by the formation of cross‐links due to cyclization and condensation. In the second step, phosphate forms and polyphosphate crosslinks with and bridges between biopolymer fragments. This produces ACs with phosphate groups. However, the produced ACs are acidic because of their phosphorus‐containing groups [159, 160, 161].

The effect of the amount of ZnCl_2_ and H_3_PO_4_ as activating agents on kraft black liquors was studied by Gonzalez‐Serrano et al. [162, 163]. In this study, the optimized precursor/activating agent ratio are reported for achieving the highest surface area of the produced ACs for ZnCl_2_ and H_3_PO_4_ activating agents, respectively. The optimized carbonization temperatures for samples activated using ZnCl_2_ and H_3_PO_4_ were reported as 500°C and 427°C, respectively. Although the obtained surface area when ZnCl_2_ was used as the activating agent was found to be greater than that when H_3_PO_4_ was used (1800/1459 m^2^ g^−1^), still H_3_PO_4_ is more preferred over ZnCl_2_ as the activating agent to avoid the toxicity of ZnCl_2_.

Different research groups have synthesized ACs using cypress (*Cupressus sempervirens*) cones as precursor to synthesize ACs via chemical activation methods using different activating agents for their application as adsorbents [100, 164]. Toscano et al. [100] first carbonized the cypress cones at 700°C and then activated it using HCl and HNO_3_ separately with a weight ratio of 1:1. After being washed, these activated samples were used as sorbents for heavy metal ions and organics. Khellouf et al. [164] used H_3_PO_4_ as an activating agent with various impregnation mass ratios from 1:1 to 1:5 to prepare AC using a spruce cone as a precursor with a carbonization temperature of 400°C–600°C. The optimal experimental conditions were a precursor‐to‐impregnating agent ratio of 1:1 and a carbonization temperature of 600°C for 4 h for the best treatment of industrial textile wastewater using the prepared AC.

In other studies, biochars and ACs were prepared using *Populus nigra* wood branches as precursor [98, 165]. Biochar was prepared by Zhang et al. [98] at different temperatures (330°C, 450°C and 600°C) to determine its effect on the adsorption of 2,4‐dichlorophenol. The maximum surface area of wood carbonized at 600°C was 334.22 m^2^ g^−1^, with a pore size of 1.97 nm; however, the best adsorption capacity was reported for biochar carbonized at 450°C because of the moderate number of pores generated in the microstructure, good electron transfer performance, and numerous functional groups. To compare their density, Ergün et al. [165] used *Populus nigra* as a precursor to synthesize ACs via the use of two different activating agents, ZnCl_2_ and H_3_PO_4_. In both cases, the chosen ratio of precursor to activating agent was 2:1, but the activation process occurred under different conditions. The precursor was treated with H_3_PO_4_ at 110°C for 2 h, while ZnCl_2_ was added to the precursor at room temperature for 24 h. Finally, the treated samples were carbonized at 600°C for 1.5 h. The density of ZnCl_2_‐activated ACs was reported to be lower than that of H_3_PO_4_‐treated ACs [165].

In any case, the reported studies are insufficient to compare the effects of the activation agent on the resulting parameters, such as surface area or pore size, for AC derived from a particular specie. Therefore, a detailed investigation of a particular specie using different activating agents is needed to comprehensively compare the effects of several activation mechanisms on the physicochemical properties of produced ACs.

##### 3.2.1.2 Impregnation Methods

The activating agent can be mixed with the biomass via the following two methods: (i) physical mixing and (ii) the impregnating method. The first consists of mixing the activating agent in its natural form (either solid or liquid) and the precursor, in a mortar and pestle or using a ball mill for a certain time at room temperature in the dry state [142, 166, 167, 168]. Instead, in the impregnation method, the activating agent is prepared in the form of a solution and mixed with the precursor for a few hours under magnetic stirring at a fixed temperature. Lillo‐Rodenas et al. [166] applied both kinds of mixing with KOH and NaOH as activating agents for two different parts of Spanish anthracite coal precursors with different ash amounts, and reported that KOH physical mixing is more effective and produces ACs with greater surface areas than other mixing methods. Physical mixing has also been attempted using ball milling in the dry state and was found to be more effective [167, 168] than the other methods.

##### 3.2.1.3 Impregnation Time

The mixing time of an impregnating agent either with biomass or with biochar is referred here as the impregnation time. It is assumed that mixing with an impregnating agent is performed to create structural pores during carbonization; therefore, the mixing time should be sufficient to guarantee that the impregnating agent can cover all the surfaces of the biomass particles and even enter into the existing open pores in the case of woody precursors. In general, proper mixing is required in the solution phase before drying it and further application of pyrolysis. As mentioned in the above section, mixing is performed using mortar and pestle or under magnetic stirring at a fixed temperature for a certain time [22, 111]. We assume that varying the impregnation time can affect the properties of the prepared ACs; however, to our knowledge, no studies have investigated its effects.

#### 3.2.2 Physical Activation

Physical activation is the mild oxidation of the precursor material with steam, CO_2_, or air at high temperatures (800°C–1000°C) [169, 170]. Both CO_2_ and steam are mild oxidants that reduce the carbon content in biochar with the creation of porosity [170]. Because of the low reaction rate between mildly oxidizing gas (steam or carbon dioxide) and biochar, a high‐temperature environment is necessary for activation [171]. In physical activation, gases are used as activating agents and are involved in a two‐step process. In the first step, the precursor is carbonized at a fixed temperature (between 600°C and 900°C) to achieve oxygen‐ and hydrogen‐free skeletons of the raw material, which results mainly in nonporous biochar. Later, in the second step, an activation process is adopted in which the biochar reacts with the oxidizing gases at a fixed temperature (between 600°C and 1200°C) to produce the final AC. This activation helps to develop more ordered micropores in ACs [42]. In particular, longer activation time helps to create more microporous structures [172]. For example, an increase in microporosity with a wide distribution of micropores was reported by Rodríguez‐Mirasol et al. [173] who prepared ACs using *Eucalyptus* kraft lignin via long physical activation (~20 h) at 850°C in the presence of CO_2_. This long activation process resulted in the creation of a reasonably high surface area (~1853 m^2^ g^−1^) with a micropore volume of approximately 0.57 cm^3^ g^−1^. In contrast, oxygen activation is preferred at relatively lower temperatures (200°C–500°C) to prevent ignition [174, 175]. Finally, simultaneous chemical and physical activation (in the presence of CO_2_) can also be achieved chemically by impregnation with dehydrating agents such as H_3_PO_4_ and ZnCl_2_ [176, 177].

### 3.3 Decomposition of Biomass during Pyrolysis

**FIGURE 7:**
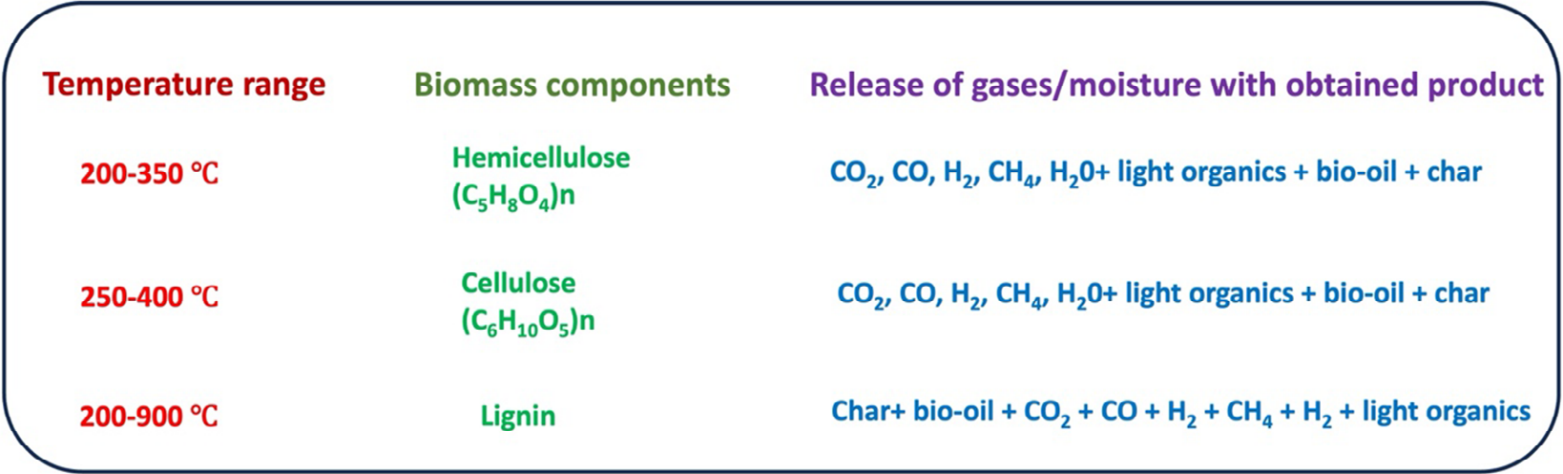
Schematic diagram representing intermediate process of biomass conversion to biogenic carbon [178].

During the pyrolytic decomposition of wood, cellulose and lignin lose non carbonaceous elements and retain the polymer skeleton with crosslinking. This process creates an aromatic sheet and a strip‐like structure, mostly bent with varying gaps on the order of molecular dimensions, referred to as micropores [41]. The degradation behaviors of cellulose, hemicellulose, and lignin differ because of their varying chemical compositions and structural variations. Hemicellulose, which encompasses units of heteropolysaccharide, decomposes first in the pyrolysis process at low temperatures, as shown in Figure 7. It has essentially an amorphous branched structure. Cellulose degrades at higher temperatures because it is more thermally stable than hemicellulose and has a sharp degradation range. Lignin decomposition occurs over a very broad range; however, the volatilization of lignin is lower than that of cellulose and hemicellulose [89, 179]. According to some studies [54, 180, 181] in which olive waste, cherry stones, and coir were pyrolyzed, compared with cellulose, lignin produces more tar than cellulose, as the authors claim that cellulose becomes volatile during pyrolysis. Therefore, the higher content of lignin in the wood precursor may increase the yield of the prepared biochar/ACs. A study on softwood and hardwood degradation revealed lower activation energies for hardwood during thermogravimetric analysis [67, 182, 183].

Among various precursors reported in the literature [11], they are mostly categorized as lignocelluloses (hard/soft wood), algae, and waste biomass. The biochar/carbon yield with composition of some examples of different kind of biomasses is reported in Table 1.

**TABLE 1:** Composition/yield values for different kind of lignocellulosic, algae, and waste biomasses.

Lignocellulosic carbon sources		Fixed carbon (%)	Composition (wt%)			References
			Cellulose	Hemicellulose	Lignin	
Lignocellulosic wood	Pine sawdust	10.6–12.2	52.52	16.4	12.6–29.3	[184, 185]
	Alder wood	21.7–23.4	32.3	23.5	24.8	[186]
	Birch wood	21.4–22.8	35.7	25.1	19.3	
	Oak wood	24.0	34.5	18.6	28.0	
	Pine wood	20.2–25.0	42.1	17.7	25.0	
	Spruce wood	22.9–25.0	41.1	20.9	28.0	
Waste lignocellulosic biomass	Coconut shell	11.9–18.8	18.7–27.2	34.1–38.0	30.2–35.1	[187, 188]
	Banana peel	0.1–14.5	40.2	10.5	24.3	[189, 190, 191]
	Wheat straw	18.83	35.0–37.5	21.2–36.0	18.0–25.7	[192, 193, 194]
	Rice husks	20.57	32.0–45.6	19.0–25.1	18.3–26.0	[194, 195]

**Algae carbon sources**		**Char yield (%)**	**Composition (wt%)**			**References**
			**Protein**	**Carbohydrates**	**Lipids**	
Algae	*Sargassum* sp	26.4–39 (500°C to 900°C)	5.45	65.5	<0.5	[196]
	*Ulva expansa*	18.9–42.7 (500°C to 900°C)	5.3	58.6	0.6	

### 3.4 Carbonization Temperature

Changes in the composition, functional properties, and pH of the final product and surface chemistry can be easily observed with varying temperature during the carbonization of a biomass. A higher carbonization temperature increases the carbon content of the biochar and decreases its hydrogen and oxygen contents because of the cleavage of weak bonds [197]. Specifically, the increase in carbonization temperature from 300°C onwards decreases the amount of volatile materials, resulting in an increase in the carbon content. Furthermore, carbonization at higher temperatures also helps to create more crystalline carbons with better mechanical properties and higher porosity.

The activation rate can increase if pyrolysis is carried out at higher temperatures, as it influences the reaction kinetics and affects the characteristics of the synthesized ACs; however, the optimal activation temperature may vary (400°C to 900°C) for different biomass precursors [198, 199, 200, 201, 202, 203, 204, 205]. Usman et al. [206] studied the effect of temperature from 300°C to 800°C on the chemical composition and the formation of elements/functional groups on biochar surfaces derived from date palm waste biomass. The carbon percentage, main cations (Ca, Mg, Na, and K), and ash amount increased with increasing carbonization temperature up to 800°C. Apart from volatiles with oxygen, the functional groups containing hydrogen, nitrogen, and sulfur also decrease with increasing carbonization temperature.

An increase in pH due to an increase in the surface basicity of the biochar has also been reported. Carbons prepared at low temperatures contain basic functional groups of the biomass; however, aromatic functional units were found to condense in the carbons produced at higher temperatures [206]. In other studies, ACs prepared at high carbonization temperatures after chemical activation using ZnCl_2_ resulted in a material with an increased carbon percentage and a lower oxygen percentage than the respective amounts contained in the precursor [165, 207, 208, 209]. This process enhances the degree of graphitization of biochar/ACs, but on the other hand, polar functional groups can detach from the biomass structure when the carbonization temperature increases [210, 211]. Different biomasses treated at the same temperature may exhibit different O/C and H/C ratios [98]. Generally, the functional groups of biomasses decrease with increasing carbonization temperature. These studies further confirm that most of the functional groups, which depend mainly on the lignin fraction, are retained in the biochar below 500°C, whereas beyond 500°C, a gradual loss of functional groups is observed up to 750°C [212].

Apart from chemical composition and surface chemistry, carbonization temperature also influences pore size, pore volume, and pore distribution in biochars/ACs. In fact, a study based on woody precursors, that is, branches of *Populus nigra*, carbonized at different temperatures (300°C, 450°C, and 600°C) revealed that the surface area increases (max of 334.22 m^2^ g^−1^ at 600°C) with increasing temperature and the pore size decreases (min of 1.97 nm at 600°C) [98]. The gradual increase in temperature creates a porous structure, which develops because of the removal of volatile substances. However, the optimum temperature to maximize the surface area of the product has been found to be dependent on the type of biomass/woody precursor [213, 214]. *Pinus ponderosa* wood and *Festuca arundinacea* grass were used to produce biochar at different carbonization temperatures in the range of 100°C–700°C [213]. The biochar of *Pinus ponderosa* wood developed a maximum surface area of 392 m^2^ g^−1^ at 600°C, whereas the biochar derived from *Festuca arundinacea* grass had a maximum surface area of 139 m^2^ g^−1^ at 700°C. Pine needle litter (*Pinus* spp.)‐derived biochar was produced at various carbonization temperatures between 100°C and 700°C and reached a maximum surface area of approximately 490.8 m^2^ g^−1^ at 700°C [214]. To obtain a well‐organized porous structure, the carbonization temperature should be at least 600°C, especially in the case of Populus nigra‐derived biochar, where a surface area of 334.2 m^2^ g^−1^ was reported in [98]. This temperature may vary from hardwood to softwood. Baklanova et al. [215] produced biochar using cedar nutshell wood as a precursor with an optimized carbonization temperature of 700°C–800°C to obtain microporous char. However, in the presence of steam activation, the effects of the carbonization temperature on the microtexture and on the other properties of produced AC were studied using hydrolytic lignin (prepared by chemical modification of native lignin)‐derived biochar. Interestingly, in the presence of steam, an increase in the carbonization temperature from 700°C to 800°C decreases the formation of micropores. In the case of ACs, the optimum carbonization temperature may tune the porosity and increase the surface area, which is critical because it is also affected by the type and amount of activating agent [63, 66]. At certain carbonization temperatures, the activation process helps to increase the pore size and surface area of the ACs. However, beyond a certain temperature, the porous skeleton may collapse, and the already developed porous structure results in a lower surface area of the ACs without a defined porous structure.

### 3.5 Heating Rate

The heating rate during carbonization directly affects the yield of synthesized AC. ACs pyrolyzed at high heating rates have lower yields than those pyrolyzed at lower heating rates do [216]. Indeed, lower heating rates promote cross‐linking in decomposed polymers along with crystallization and a slower volatilization of organic molecules from the condensed phase of the material, increasing the yield [62]. Biomass degradation is favored at temperatures above ~500°C because of the release of volatile fractions and therefore results in lower char yields. Kazimierski et al. [216] synthesized ACs using slow and fast heating methods (15°C min^−1^ and 100°C min^−1^) at three different carbonization temperatures for three different precursors: apple (*Malus domestica*), pear (*Pyrus communis* L.), and plum (*Prunus domestica* L.). Their results suggested that at 400°C, the highest yields were observed with slow pyrolysis, whereas the lowest yields were recorded at 600°C when fast pyrolysis was used. Their results clearly suggest that high char yields (with a lower percentage of carbon content) can be obtained by applying slow heating rates during pyrolysis at lower temperatures.

### 3.6 Functionalization/Doping

Surface modification is another important method that can be used to prepare potentially more useful ACs for electrochemical and energy storage applications. During impregnation or later, the use of various elements, such as N, S, P, and B, is preferred for obtaining functional groups on the surface of ACs. As mentioned earlier, woods have microchannel walls that can be easily transformed into different structures with modifications in chemical composition and functionalization. The amount of functionalization depends on wood characteristics and operating conditions and creates more adsorption centers/active sites, which help to adsorb ions and gas molecules on the surface of ACs [217, 218, 219]. Organic pollutants can bind to biochar surfaces through *π*—*π* bonds, even without the functionalization of AC surfaces [220]; however, it becomes more important to use biochar as an inorganic pollutant absorbent. Metal incorporation in the AC matrix has been proven to increase the anionic contaminant sorption capacity [221, 222]. The large incorporation of oxygen‐containing functional groups on biochar/AC surfaces helps to increase the number of polar groups and carbon hydrophilicity, which makes them more useful for adsorption applications [39, 223].

Doping is also used to improve the electrochemical properties of WDACs. Nitrogen doping (N‐doping) creates defects and functional groups in woody carbon skeletons, such as pyridinic‐N, pyrrolic‐N, and graphitic‐N, making it suitable for applications in electrochemical devices as a current collector and catalyst [30, 223, 224]. The oxygen reduction reaction (ORR) generally is slow because of the strong O—O double bond, which affects the reaction kinetics and catalytic properties of carbon‐based cathodes [224]. Owing to the high electronegativity of nitrogen, N‐doped carbons redistribute the charge of carbon atoms, generating a positive charge density, and therefore changing the chemisorption of oxygen by weakening O—O bonds [225, 226]. N‐doped woody ACs are strongly hydrophilic [227], present defect sites because of heteroatom doping, and can trap electrolyte ions more efficiently [228]. Therefore, N‐doped ACs have shown enhanced electrochemical properties in the ORR similar to those of platinum; therefore, they are being investigated for their ability to replace noble metal‐based electrodes.

### 3.7 Inert Gas and its Flow Rate

Injection of an inert gas in the reactor during carbonization is required to avoid the presence of oxygen and to remove volatile compounds produced during pyrolysis from the sample. The flow of inert gas, for example, nitrogen gas, inside the reactor during carbonization reduces the residence time of pyrolysis vapors and prevents their repolymerization. Another advantage is that only hot vapors are removed from the reaction zone [229]. The flow rate of inert gas influences the yield and physicochemical properties of the prepared ACs. By increasing the flow rate, volatile particles can be removed faster from the material surface; therefore, secondary deposition of the volatiles can decrease significantly, increasing the fixed carbon content. Lua et al. [230] studied the effects of varying nitrogen flow rates on the preparation of biochar and ACs using pistachio nutshell as a biomass. In the case of biochar, with increasing nitrogen flow rate, the fixed carbon content increased because of the decrease in the volatile matter content. Afterwards, the ACs were prepared using chars; therefore, even with increasing nitrogen flow rate, the amount of fixed carbon remained constant for all the chars [230]. The inert gas flow rate also affects the pore development and surface area of a sample [231, 232]. Bouchelta et al. [231] and Lua et al. [232] studied the effect of the N_2_ flow rate on the development of the porous structure in ACs produced using date pits and oil‐palm shells, respectively. They reported that an increase in the gas flow rate up to ~150 cm^3^ min^−1^ increased the micropore volume to 0.215 cm^3^ g^−1;^ therefore, the surface area of the ACs increased to 19 m^2^ g^−1^. Beyond the optimal flow rate (300 cm^3^ min^−1^), they reported a decrease in pore volume because of the high N_2_ flow rate, which decreased the temperature of the char particles and resulted in a lower reaction rate, which may have reduced the release of volatiles and resulted in decreased pore formation.

Although these studies are performed on PBBs as precursors, similar effects are expected in the case of woody precursors.

### 3.8 Washing of Carbon

High‐temperature pyrolysis of woods helps to produce stable disordered carbon structures. However, in this process, solid by‐products with tar are also formed, which remain attached to the surface of the ACs or inside the created pores. Additionally, biochar or ACs produced at high temperatures from wood precursors are generally highly alkaline because of the presence of inorganic carbonates [233]. In addition, a greater ash content and more positive charges formed on the carbon surface during activation are responsible for increasing the pH [147]. Therefore, it is important to remove the ash and bio‐oil contained in the pyrolyzed sample and neutralize the final carbon before it is used in an application. Different types of washing are used, mainly using water or other chemicals such as acids and ethanol. After being washed with a solvent, repeated double‐distilled water washing helps to clean the carbonized samples and maintain a neutral pH [22]; however, it also helps to remove ashes present in the form of sand, dust, or other organic impurities.

#### 3.8.1 Washing with Acids

Acid washing helps to dissolve inorganic compounds present as impurities in ACs, such as metal oxides, and other organic contaminants, such as ash, tar, and residual hydrocarbons, and helps to open closed pores. However, highly concentrated acids may etch the surface and collapse the pore structures, while oxidation with strong acids may also decrease the thermal stability of ACs. Acid washing may introduce oxygen‐containing functional groups that depend on the chosen acid (e.g., hydroxyl, carboxyl, or carbonyl). The catalytic properties and adsorption of polar molecules by ACs can be enhanced by attaching sulfonic or phosphate groups, which can be introduced using H_2_SO_4_ or H_3_PO_4_ as chemical agents. The introduction of sulfonic groups can also enhance the hydrophilic nature of AC [234]. Instead, less aggressive acid washing is appropriate for maintaining the original carbon structure because harsh acid washing may reduce the electrical conductivity through excessive oxidation. Understanding the impact of the selected acid for washing is important, as acids can affect the thermal stability and other properties of synthesized ACs. High concentrations of acids may have a strong effect on ACs. H_3_PO_4_ can produce more functional groups and create further pores in the carbon structure [235]**.** Therefore, if pores are already optimized during activation, washing of the ACs with H_3_PO_4_ is not suitable. However, compared with other acids such as HCl, H_2_SO_4_, and HNO_3_, a 1 M solution of H_3_PO_4_ has been reported to be better for the formation of extra pores in already prepared ACs [236]. In fact, 1 M H_3_PO_4_‐treated commercial ACs were assessed as cathodic materials for microbial fuel cells with a high power density of ~1546 mW m^−2^ [236].

Generally, 0.1 M to 1 M HCl solutions are used to remove ash from the prepared ACs and, in particular, to open the pores formed in the activation process, improving the overall quality of the ACs. Low‐concentration HCl washing does not completely alter the surface chemistry of prepared ACs; however, single‐bonded O‐functional groups (ethers, phenols, and lactones) are attached to the surface of ACs during HCl washing [235].

Pyrolyzed carbons are also used with aqueous electrolytes during their application in energy devices. To achieve efficient reactions, the hygroscopic surface of the carbon helps to increase the wettability of the carbon surface with the electrolyte. The immersion of ACs into a 1 M HNO_3_ solution at 150°C for three to 4 h can alter the chemical nature of the carbon surface and can help in the grafting of hydroxyl, carboxyl, and nitrate groups on its surface [237]. The residual HNO_3_ can be removed after hot water washing under sonication [65, 235]. Finally, after being washed with double‐distilled water and dried in an oven, the carbon can be used for appropriate applications. Nevertheless, a higher concentration of HNO_3_ can adversely influence the carbon structure because it can alter the structure of pores or even collapse micropores [237].

#### 3.8.2 Washing with Ethanol

Ethanol washing helps to remove oil, residual tar, and other organic‐by‐products formed during pyrolysis. Excess ethanol washing can etch the carbon surface and reduce its acidic/polar functional groups, which may affect the conductivity of the AC. Generally, ethanol washing does not alter the overall structure of the ACs [238]. High ethanol concentrations with long washing times can rearrange the surface layer and can cause swelling in the sample. Although the ethanol washing effect is not very notable, care must be taken, especially in terms of reducing the washing time to avoid affecting the internal surface and overall structure of the prepared carbon. Ethanol can dissolve low‐molecular‐weight compounds; therefore, it may increase the porosity and surface area of the ACs.

### 3.9 Porosity and Surface Area

The regular porous structure and optimized number/size of pores are important factors for the application of ACs, especially in energy devices. As mentioned earlier, natural wood itself has a porous structure. Pores smaller than 25 µm can be easily obstructed, increasing the charge transfer resistance during their use in electrochemical devices; however, the presence of more macropores reduces the number of effective sites [29], and therefore, it is necessary to tune the wood porosity while it is being processed into AC. Considering the presence of various kinds of pores in AC, the key factor for improving its performance is controlling the ratio of effective porosity to average pore size. ACs prepared using the lignin component of softwood and hardwood under the same experimental conditions have been shown to have different morphological structures [150], confirming that the wood structure strongly affects the final properties of synthesized ACs. The hardwood‐derived lignin‐based ACs had rough surfaces, irregular pores, uniform crack diameters, and meso‐ and macroporous structures. However, softwood‐based lignin was reported to possess a hierarchical porous structure with the presence of honeycomb‐shaped pores. Other studies [22, 39, 40] have confirmed that the wood structure strongly influences the physicochemical properties of biochar, which may be modified by the activation process.

The development of an effective pore structure largely depends on the choice of wood precursor, as well as on the activation and carbonization methods employed and the presence of additional supporting materials. Yu et al. [29] fabricated gas diffusion electrodes (modified biochar) using cherry (*Prunus avium*), oak (*Quercus*), and pine (*Pinus sylvestris*) wood as biomass precursors through an identical preparation procedure; nevertheless, the resulting ACs exhibited markedly different pore size distributions. Cherry‐derived ACs were predominantly enriched with macropores averaging ~45 µm, oak‐derived ACs exhibited larger macropores exceeding 100 µm, whereas pine‐derived ACs displayed significantly smaller pore sizes (~15 µm) compared to the other two [29]. The activating agent is another critical factor governing the development of the porous architecture. Different chemical activators can yield ACs with distinct pore sizes and volumes, even when derived from the same wood precursor [58, 165, 239]. Ergün et al. [165] synthesized AC from poplar wood (*Populus nigra*) using ZnCl_2_ and H_3_PO_4_ as activating agents and observed markedly different morphologies between the two samples. ZnCl_2_‐ACs exhibited hollow spherical pores with smooth walls and variable inter‐pore spacing across the surface, whereas activation with H_3_PO_4_ resulted in the formation of elliptical pores.

The porosity, pore type, and surface area can be estimated using nitrogen absorption isotherms and BET analysis. The pore size distribution can also be measured from nitrogen desorption isotherms. The high slope of nitrogen isotherms at very low relative pressure and nitrogen absorption saturation at high pressures suggest the formation of large amounts of micropores, which are typical of type I isotherms. However, an increase in nitrogen absorption with increasing relative pressure indicates the presence of micropores, mesopores, and macropores. Many factors can affect the existence of these different kinds of pores in ACs. Reports have shown that an increase in the carbonization temperature creates larger pores [172]. The volume ratios for different kinds of pores (*V*_micro_/*V*_meso_/*V*_macro_) with respect to the total pore volume *V*_total_ help to calculate the respective amounts of pores present in a synthesized AC.

Pore volume and pore size are parameters used to describe the morphology of an AC. However, absolute pore volume is another important parameter, mainly for industrial purposes, which helps to estimate the highest amount of micropore volume from a specific amount of biochar [55]. The absolute pore volume can be calculated as follows:

$$\text{Absolute}\;\text{pore volume}=\frac{\left(\text{pore}\;\text{volume}\times \text{final}\;\text{weight}\;\text{of}\;\text{AC}\right)}{\text{initial}\;\text{weight}\;\text{of}\;\text{used}\;\text{biochar}}$$

The variation in the absolute micropore volume as a function of increasing burn‐off has been reported in few studies [55, 240, 241]. This study revealed that the maximum absolute micropore volume occurred for each burn‐off value. From this study, one can easily estimate how much burn‐off is required to create maximum micropores for a fixed amount of biochar. However, the value of the obtained maximum micropore volume for a particular biochar can vary as a function of the activation method and activating agent/gas.

### 3.10 Amorphous/Graphitic Structure

ACs prepared through pyrolysis generally exhibit both graphitic and disordered natures. Structural information on ACs can be obtained via XRD and Raman spectroscopy studies. The graphite content in ACs is related to their electrical conductivity. A graphitic/ordered structure is already present because of the cellulose contained in the wood, whereas the amorphous structure is derived mainly from hemicellulose and lignin [50, 65, 81, 242, 243]. As the cellulose/lignin content depends on the chemical composition of the wood, the D and G bands in the Raman spectra differ, where the D band intensity represents the presence of defects (disordered graphitic sp^3^ structure)/amorphous regions and the G band intensity provides information about the ordered crystalline graphite featured regions in the ACs. It is possible to enhance the graphitic or amorphous nature of the ACs by properly tuning the carbonization temperature. At higher carbonization temperatures, ACs have more carbon content with respect to hydrogen or oxygen; therefore, the degree of graphitization is increased [6]. Apart from the D and G bands, in most cases, additional D* (~1220–1270 cm^−1^) and D″ (1520 cm^−1^) bands are also present in the Raman spectra of WDACs [104]. The D* band refers to amorphous carbon related to the D band and C—C vibrations of cis‐polyacetylene groups [244]**,** whereas D″ is related to the amorphization of ACs [245, 246, 247, 248, 249] and is also related to the C=C vibration of polyenes [250, 251].

**FIGURE 8:**
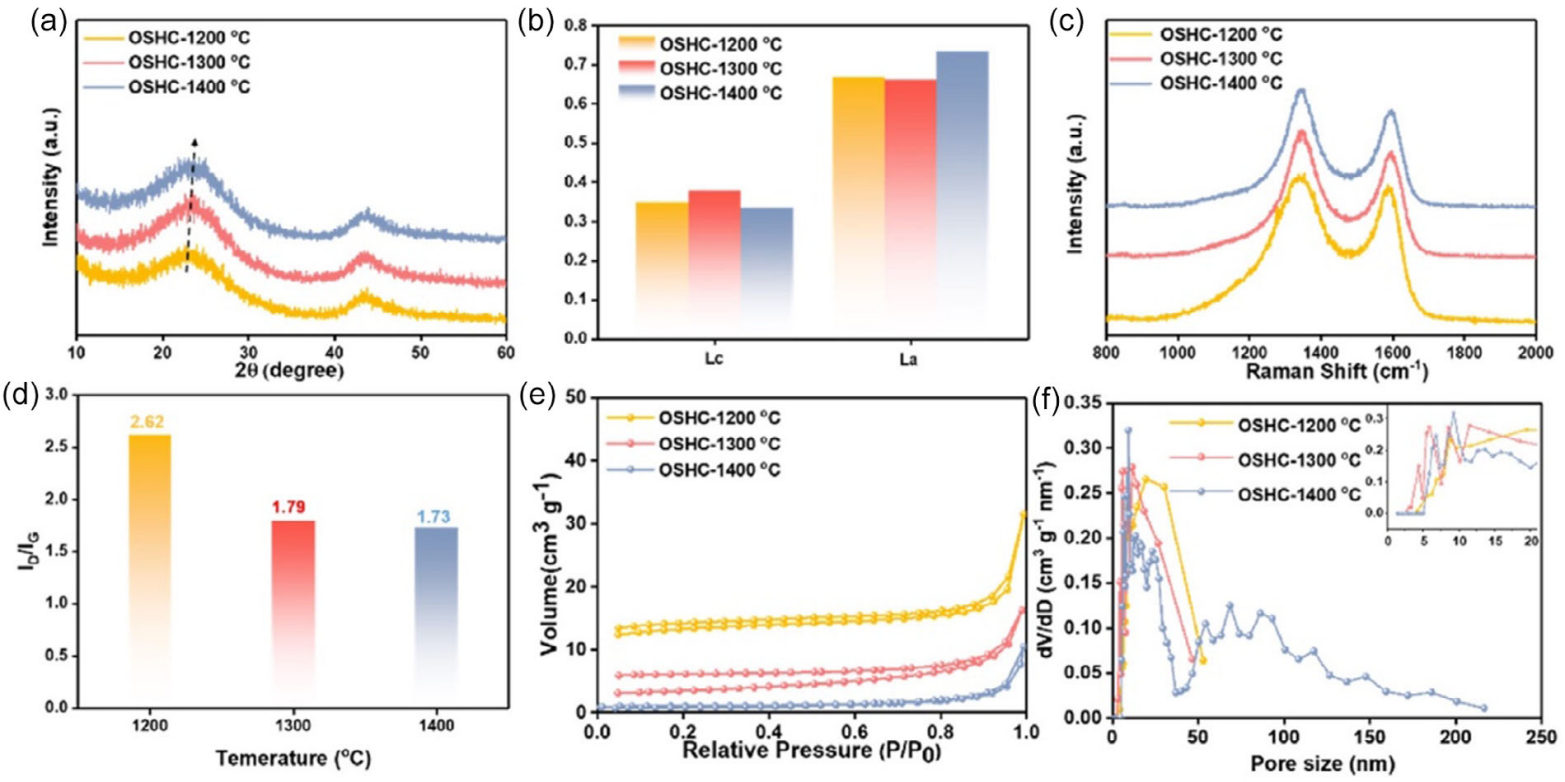
Effect of temperature on the structural and surface properties of the carbons shown through (a) XRD graphs, (b) Lc and La values, (c) Raman shift, (d) ID/IG values, (e) BET isotherms, and (f) PSD. Reproduced from ref. [252]. Copyright (2025), Elsevier B.V.

The degree of graphitization and interlayer spacing are key structural factors determining performance. A higher graphitization degree enhances electrical conductivity and rate capability, whereas appropriate interlayer spacing facilitates metal‐ion storage. As temperature increases, graphitization improves but interlayer spacing decreases. Zhon et al. [252] pyrolyzed olive shell biomass at 1200°C–1400°C, finding that carbon prepared at 1300°C exhibited optimal Na‐ion battery performance due to enhanced ion diffusion channels. The effect of temperature on created structure of the carbon materials is shown in Figure 8 [252].

High electrical conductivity minimizes charge–transfer and diffusion resistances, essential for energy devices. However, pristine biochar often exhibits low graphitization and conductivity, necessitating activation or heteroatom doping. Wang et al. [253] produced nitrogen‐doped and KOH‐AC from *Acacia confusa* wood, achieving a transition from dense to porous veil‐like morphology and improved graphitic ordering and conductivity. In particular, conductivity was found to increase from 0.11 S cm^−1^ (biochar) to 9.37 S cm^−1^ (AC). The AC electrode delivered a specific capacitance of 129.0 F g^−1^ and 90% retention over 30 000 cycles in an organic electrolyte of tetraethylammonium tetrafluoroborate with propylene carbonate (1 M TEABF_4_/PC), outperforming than commercial porous carbons.

## 4 ACs from Biomass Materials

Most ACs exhibit pseudomorphs of the original structure of the precursor used [41]. The original texture of the wood cells of any plant is responsible for the final morphology and microstructure of the prepared ACs and is usually unaffected by carbonization or activation processes, if the precursor does not pass through a fluid stage [41]. However, chemical and structural differences and differences in the proportions of the main components of wood (cellulose, hemicellulose, and lignin), and the presence of different extractives, and other non‐structural substances influence their thermal behavior; therefore, significant differences are observed in the pyrolysis of softwood and hardwood [67]. The presence of different amounts of cellulose and lignin affects the microstructures of prepared ACs [243]. There may be different amounts of shrinkage in the axial, radial, and tangential directions of wood, but the wall structure remains protected during heat, chemical, or gas treatments [62]. Differences in cellular components and their variable chemical characteristics between hardwood and softwood result in different microstructures, specific surface areas, and active sites in the produced ACs [86]. Hardwood is preferred for creating all kinds of pores (macro‐, meso‐, and micropores) in ACs [172]. Although the presence of large pores in ACs produced from hardwoods can further increase via carbonization at higher temperatures, it also depends on other synthesis factors [172]. Most studies have demonstrated that ACs produced from softwoods may exhibit a higher number of micropores [172] and therefore a lower surface area [97, 242, 254]; in contrast, more meso‐ and macropores can be generated during hardwood pyrolysis. Even higher carbonization temperatures do not help to create meso‐ or macropores in ACs produced from softwoods.

The activation rate for a material depends on its chemical composition, especially on the cellulose‐to‐lignin ratio and its porosity. Materials with high cellulose‐to‐lignin ratios and high porosity can be activated easily [55]. Many pores increase the surface area of the precursor, which leads to a better activation rate. However, Daud et al. [55] reported that a higher porosity of the precursor does not ensure a higher activation rate. It is possible that a less porous material may have high activation rates because of its cellulose content is greater than that of its lignin [55]. The gradient of the curve between the activation time and burn off represents the activation rate [55]. Here, the burn off represents the percentage of material loss after carbonization with respect to the initial mass of the precursor. A comparison of activation rates for fibrous‐structured coconut shell char and less fibrous palm shell char was performed by Daud et al. [55]. Coconut char has a much higher activation rate, nearly 5 times higher than that of palm shell char, and this difference was attributed to its lignocellulosic components. More cellulose and halocellulose contained in coconut shell resulted in a lower activation rate than in palm shell char, which has a higher lignin content. The results may also be generalized to woody precursors because of their similar constituents. Therefore, the activation process can be easier for hardwoods, as they contain more cellulose than lignin [55]; however, a longer activation time is required for soft carbons containing more lignin [55, 173].

Biochars/ACs derived from different biomasses demonstrate distinct hydrogen‐to‐carbon ratios (H/C), which depend on pyrolysis conditions. Biochars/ACs with lower H/C ratios have better carbonization and carbon stability, especially in the presence of aromatic carbon [255]. Instead, a high H/C ratio represents weak carbonization with less stable carbons. Jiang et al. [39] prepared biochar from hardwood (jarrah) and softwood (pine), and reported that hardwood‐based biochar was more stable and easier to carbonize.

Some studies have investigated the production of ACs using softwood and hardwood precursors under the same experimental conditions [39, 65, 150, 172, 256]. In other studies, comparisons have been made between the ACs produced using the lignin component of softwood and hardwood [150, 256], mixtures of different woods [24, 256] and using whole wood itself as a precursor [39, 65].

### 4.1 Softwood/Lignin‐Based Precursors for ACs

As mentioned above, ACs synthesized using softwood are known to have lower surface areas, and more micropores. However, ACs synthesized from todo fir [131] and a mixture of Norway spruce (*Picea abies*) and scots pine (*Pinus sylvestris*) [132] could present BET surface areas more than 1000 m^2^ g^−1^, which can be attributed to physical carbonization effects. A comparison of these studies revealed that physical activation is more effective than chemical activation; however, if both are applied together, the surface area can be further enhanced [131, 132]. The microstructures of the ACs synthesized using different softwoods from different research groups are compared in Table 2.

**TABLE 2:** Microstructure analysis comparison and pyrolysis parameters of ACs derived from various softwood species.

Wood species	(a) Used chemical/physical activating agent (b) Doping agent	Ratio of biomass/activating agent	(a) Carbonization temperature (°C) (b) Residence time (hours)	Total surface area (m^2^ g^−1^)/Microsurface area (m^2^ g^−1^)	Micropore volume (cm^3^ g^−1^)/mesopore volume (cm^3^ g^−1^)	Total pore volume (cm^3^ g^−1^)	Pore size (nm)	Application	References
Date palm waste (leaves + branches + stem barks)	(a) None (b) None	—	(a) 300 (b) 4	—	—	—	—	—	[206]
			(a) 400 (b) 4						
			(a) 500 (b) 4						
			(a) 600 (b) 4						
			(a) 700 (b) 4						
			(a) 800 (b) 4						
Todo fir (*Abies sahalinensis*)	(a) Steam activation at 900°C (for 40 min to 120 min) (b) None	—	(a) 1000 (b) 1	1560 (maximum)	—	—	—	Adsorption properties (capacity to methane)	[131]
*Pinus sylvestris*	(a) CO_2_ activation at 800°C for 3 h (after first carbonization) (b) Pd decorated	—	(a) 600 (b) 3	412	—	—	—	Catalytic reduction of 4‐Nitrophenol	[65]
Cladodes (*Opunita ficus indica* L.)	(a) 12% NaOH solution (pretreatment) and H_3_PO_4_ (activating agent) (b) None	1:2	(a) 600 (b) 1	—	—	—	—	Adsorbent for anionic dye Red Bemacid	[257]
Norway spruce (*Picea abies*) + Scots pine (*Pinus sylvestris*)	(a) ZnCl_2_ (b) None	1:1	(a) 600 (b) 1	1217/602	—	0.27	—	Adsorption	[132]
	(a) ZnCl_2_ (b) None		(a) 800 (b) 1	1018/528	—	0.24	—		
	(a) First steam explosion then ZnCl_2_ (b) None		(a) 600 (b) 1	1308/662	—	0.30	—		
	(a) First steam explosion then ZnCl_2_ (b) None		(a) 800 (b) 1	1217/682	—	0.29	—		
Pine Bark (*Pinus sylvestris*)	(a) H_3_PO_4_ (b) None	1:5	(a) 600 (b) 2	8.79/7.49	—	0.3302	2.05	Supercapacitor	[99]
			(a) 800 (b) 2	388.67/355.46	—	0.3307	1.61		
			(a) 1000 (b) 2	583.06/−529.36	—	0.3297	1.64		
	(a) K_2_CO_3_ (b) None	1:5	(a) 600 (b) 2	527.22/529.48	—	0.3283	1.85		
			(a) 800 (b) 2	743.3/746.27	—	0.9940	1.87		
			(a) 1000 (b) 2	1016.93/973.8	—	0.3274	1.68		
	(a) ZnCl_2_ (b) None	1:5	(a) 600 (b) 2	673.12/671.63	—	0.3293	1.59		
			(a) 800 (b) 2	706.94/666.48	—	0.3287	1.59		
			(a) 1000 (b) 2	709.33/737.67	—	0.9936	3.12		
*Cupressus sempervirens* L	(a) HCl (b) None	1:1	(a) 700 (b) 2	510/—	—	—	—	Adsorption performance	[100]
	(a) HNO_3_ (b) None			420/—					
*Cupressus sempervirens* L.	(a) H_3_PO_4_ (b) None	1:1	(a) 400 (b) 4	—	—	—	—	Adsorption of methylene blue dye	[164]
			(a) 600 (b) 4						

### 4.2 Hardwood/Cellulose‐Based Precursors for ACs

As mentioned in Section 3, compared with softwood, ACs synthesized using hardwood have a higher surface area and larger pore size. ACs derived from *Eucalyptus* wood [258], birch (*Betula pendula*) wood [104], and teak wood [259] have high surface areas of 1817, 2926, 2138.3, and 1345.25 m^2^ g^−1^, respectively. Comparisons of the microstructures of the ACs synthesized using different hardwoods by different research groups are presented in Table 3.

**TABLE 3:** Microstructure analysis comparison and production parameters of ACs derived from various hardwood species.

Wood species	(a) Used chemical/physical activating agent (b) Doping agent/extra treatment	Ratio of biomass/activating agent	(a) Carbonization temperature (°C) (b) Residence time (hours)	Total surface area (m^2^ g^−1^)/Micro surface area (m^2^ g^−1^)	Micropore volume (cm^3^ g^−1^)/mesopore volume (cm^3^ g^−1^)	Total pore volume (cm^3^ g^−1^)	Pore size (nm)	Application	References
*Eucalyptus*	(a) KOH (b) None	—	(a) 800 (b) —	1817/—	—	0.84	—	Supercapacitor	[258]
Birch (*Betula pendula*)	(a) NaOH (b) Nitrogen	1:3	(a) 700 (b) 2	2909/—	0.92/0.74	1.67	—	ORR	[104]
Teak (*Tectona grandis*)	(a) KOH (b) Later microwave treatment	1:1.15	(a) 550 (b) 1	1345.25/878.63	—	0.6140	2.85	Dye removal	[259]
*Populus nigra*	(a) ZnCl_2_, (b) None	2:1	(a) 600 (b) 1.5	—	—	—	—	—	[165]
	(a) H_3_PO_4_ (b) None			—	—	—	—	—	
Tamarind (*Tamarindus indica*)	(a) ZnCl_2_ (b) None	1:1	(a) 439 (b) 40 min	1322/—	—	1.042	1.06	Wastewater treatment	[260]
*Populus nigra*	(a) — (b) —	—	(a) 300 (b) 2	2.45/—	—	—	17.23	Adsorption of 2,4‐dichlorophenol	[98]
			(a) 450 (b) 2	61.85/—	—	—	3.29		
			(a) 600 (b) 2	334.22/—	—	—	1.97		
Poplar NE222 (*Populus*)	(a) H_3_PO_4_ (b) —	—	(a) 450 (b) 1.5	2015	0.19/0.73	1.2	2–8	Dye adsorption	[261]
Acorns (*Quercus* spp.)	(a) HCl, (b) None	1:1	(a) 700 (b) 2	560/—	—	—	—	Adsorption performance	[100]
	(a) HNO_3_ (b) None			440/—					
Plum stones (*Prunus domestica* L.)	(a) 2M MgCl_2_ (b) None	200 mL of 2 M MgCl_2_ for 10 g of precursor	(a) 500 (b) 1.5	—	—	—	—	None	[262]
Basswood (*Tilia americana*)	(a) CO_2_ activation at 800°C for 3 h (after first carbonization) (b) Pd decorated	—	(a) 600 (b) 3	474	—	—	—	Catalytic reduction of 4‐Nitrophenol	[65]
*Quercus variabilis* (Cork)	(a) H_3_PO_4_ (b) N, P, and O	1:0.5	(a) 600 (b) 1	364.26	0.15/0.04	0.19	1.07	Supercapacitor	[263]
		1:1		515.80	0.21/0.08	0.29	1.40		
		1:2		446.99	0.18/0.08	0.26	1.56		
		1:3		404.52	0.17/0.04	0.21	1.76		

Magnesium chloride: MgCl_2_.

### 4.3 Other Biomass‐Based Precursors for ACs

Apart from wood, other biomasses have long been employed as precursors for the synthesis of ACs because of their easy availability and easy initial preparation. ACs prepared using biomass precursors mainly contain a fine pore distribution of only a few nanometers, as in the case of coconut shell, the pore radius was reported to be less than 1 nm; however, ACs derived from woody precursors contain a variety of mesopores and micropores [264]. The use of raw biomass as a precursor influences the pore shape and pore size distribution [265]. The microstructures of the ACs synthesized from different biomasses from different research groups are compared in Table 4.

**TABLE 4:** Microstructure analysis comparison and production parameters of ACs derived from other biomasses.

Used biomass	(a) Used chemical/physical activating agent (b) Doping agent	Ratio of biomass:activating agent	(a) Carbonization temperatures (°C) (b) Residence time (hours)	Total surface area (m^2^ g^−1^)/Micro surface area (m^2^ g^−1^)	Micropore volume (cm^3^ g^−1^)/mesopore volume (cm^3^ g^−1^)	Total pore volume (cm^3^ g^−1^)	Pore size (nm)	Application	References
Fruit shells of *Sterculia foetida* (SF)	(a) KOH (b) —	1:3	(a) 700, 800, 900 (b) 2	713–1040.5	—/0.295	0.172 to 0.295	2.202 to 2.233	Supercapacitor	[101]
Camphor tree grains	(a) 16 M Nitric acid (b) —	—	(a) 800 (b) 2	470.5/—	—	0.798	1.67		[143]
	(a) Copper chloride (b) —	1:10		1754.5/—	—	0.196	1.82		
Pomelo peels	(a) KOH (b) —	1:4	(a) 800 (b) 2	950.490/776.35	0.40/—	0.51	2.52		[102]
		1:5		1277.14/1028.17	0.54/—	0.69	2.35		
		1:6		1190.75/884.75	0.46/—	0.65	2.39		
	(a) KOH (b) Melamine	1:5 and doping ratio: 2:1	—	—	—	—	—		
*Eucommia ulmoides Olive*r	(a) H_3_PO_4_ (b) Hydrothermal treatment at 170°C before impregnation	1:4	(a) 400 (b) 6	2138.3/587	0.26/0.83	1.09	—	Supercapacitor	[266]
Seeds of *Jatropha curcas*	(a) H_3_PO_4_ (b) None	1:2	(a) 500 (b) 2	—	—	—	—	Water absorption capacity	[267]

## 5 ACs Derived From Biomasses for Use in Electrochemical Applications

ACs derived from agricultural waste are cost‐effective green nanomaterials that are suitable for various applications, such as waste recycling [43], controlling air pollution [268], treating wastewater [269], enhancing soil fertility [270], renewable energy [271], and biotechnology [272]. Supercapacitors, batteries, dye extraction from industrial wastewater, and fuel cells are the main applications and exhibit unique advantages and disadvantages, which are being addressed by different research groups.

ACs derived from different wood types have different properties in terms of density, porosity, and conductivity. Both gravimetric and volumetric densities affect the performance of supercapacitor/batteries, and their balance is critical during synthesis. In terms of porosity, the presence of mesopores is more useful for supercapacitors; however, determining which suitable biomasses can provide what types of pores remains a matter of in‐depth study. Highly porous carbons are low‐density carbons and therefore provides high gravimetric density [273, 274]. Owing to the presence of a high number of pores, the surface area of the material increases, and greater charge is stored with the same amount of material, which increases its gravimetric density. Nevertheless, determining what kind of biomass and which synthesis methods can create a balance between gravimetric and volumetric density by creating optimum pores remains a research problem.

The incorporation of active sites and the high conductivity of ACs make them more suitable candidates for energy device applications than transition metal oxides, which generally suffer from low conductivity and high costs [275, 276].

### 5.1 ACs in Batteries

The demand for batteries is to obtain high energy density without sacrificing sufficient capability because of their use in disposable portable electronic devices. To increase the energy density of cells using porous cathode materials, a thick layer of high‐density cathodes is needed until the accessible capacity is limited because of insufficient ion transport through a small volume of tortuous pores [277, 278]. The tortuosity (τ) of any porous electrode material is the length ratio, which can be calculated as follows [279]:

$$\tau =\frac{\text{microscopic}\;\text{path}\;\text{length}\;\text{travelled}\;\text{by}\;\text{ion}\;\text{within}\;\text{the}\;\text{pores}}{\text{Cartesian}\;\text{distance}\;\text{between}\;\text{the}\;\text{endpoints}\;\text{of}\;\text{the}\;\text{path}}$$

If the τ of a material is greater than one, this indicates random arrangements of an impenetrable matrix within the electrode [279]. Clearly, ordered vertical pores are required in a structure for fast ion transport during functioning of the cell, providing optimal τ. Therefore, the structure of an efficient cathode material requires low τ with many pores. Such a structure provides active sites with fast ionic transport to the species responsible for reactions and provides efficient electron transfer within the whole electrode structure [280].

Lithium‐ion batteries (LIBs) are mostly developed as energy storage devices but have safety issues. Metal‐air batteries (MABs) based on gas consumption reactions are another kind of emerging battery. Among different metal air batteries, aluminum air batteries (AABs) are gaining attention because of their safety and because of their high theoretical energy density of ~8100 Wh kg^−1^ (referred to the anode) [281]. Additionally, the low weight and abundant nature of aluminum explain its use in AABs. In an AAB, aluminum is used as an anode material, and AABs exhibit a simple construction process. The performance of AABs depends mainly on the nature of the electrolyte used with the air cathode material. The catalytic layer, gas diffusion layer (GDL), and current collector together comprise air cathodes with specific functions. The GDL must have optimum pores to perform ORR while avoiding water collection; otherwise, oxygen diffusion pathways will be restricted [282]. The ORR is promoted by the catalyst layer through four‐electron transfer pathways. Two important factors for working as a suitable air cathode are its intrinsic activity and ability to provide low mass transfer resistance for gas species. Platinum is the most efficient cathode but is a costly option, and other inexpensive transition metal oxides are not very safe options for use as cathodes. Commercial carbon papers are costly at ~1400 $ m^−2^ and are fabricated using short carbon fibers that are randomly knitted [29] and therefore offer random gas diffusion channels that increase τ. Wood‐based cathodes have been proven to be the best air cathodes because of their original ordered structure with multiple microchannels. Materials embedded with properties such as diffusion of ions, corrosion resistance, and desired gas transportation properties are required as cathode materials in batteries, and these properties can easily be offered by WDACs because of their porous and less tortuous structure. Therefore, WDACs are also known as “breathable materials”. The use of WDACs as anodes and cathode catalysts in batteries is being attempted to make the system cost‐effective and at present to increase its performance. The addition of noble metals to ACs is an option for researchers, however, this increases the cost of the electrodes. In a particular application, ash, pine, and oak wood‐based GDEs ensure the feasibility of AABs, and the key performance metric is the ratio of the effective porosity to the average pore size [29].

**FIGURE 9:**
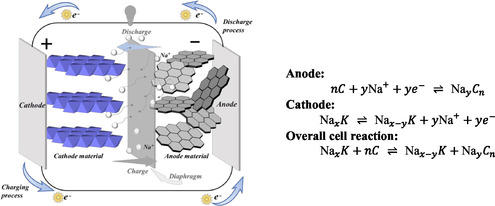
Schematic of the working mechanism of sodium‐ion battery. Reproduced from ref. [286]. Published by MDPI.

**FIGURE 10:**
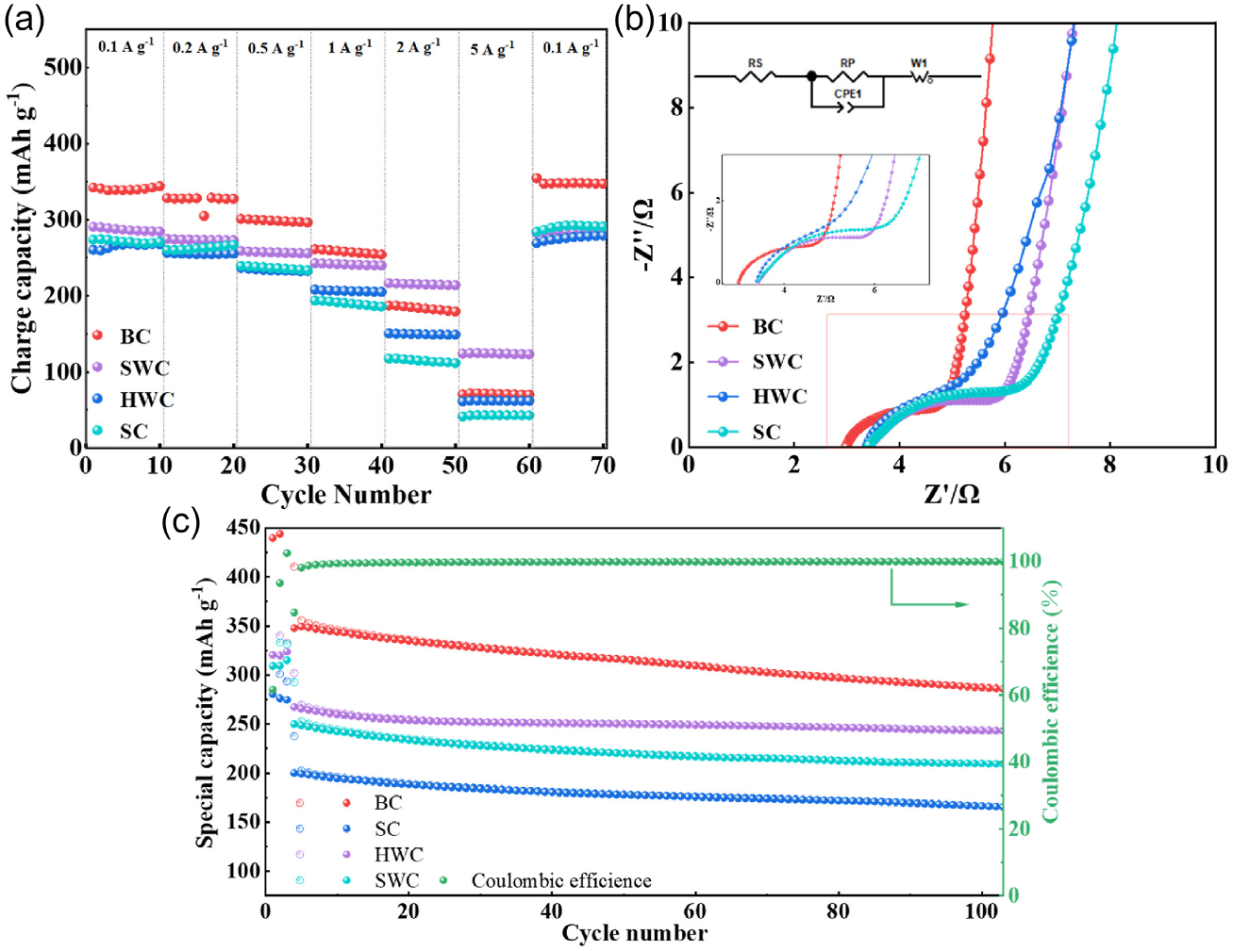
Performance graphs of sodium‐ion batteries using different hardwood‐derived carbons: (a) charge capacity vs. cycle number; (b) impedance patterns with electrical parameters; and (c) capacity vs. cycle number. Reproduced from ref. [287]. Copyright (2025), Elsevier B.V.

Sodium‐ion batteries (SIBs) represent an important additional class of electrochemical systems that benefit from carbon anodes specifically tailored for reversible Na^+^ storage [283]. Their operation follows a typical “rocking‐chair” mechanism, in which sodium ions shuttle between a carbon anode and a cathode featuring open intercalation frameworks [284, 285]. During discharge, Na^+^ ions are released from the anode, migrate through the electrolyte, and intercalate into the cathode structure; during charge, the process occurs in the reverse direction, as illustrated in Figure 9 [286]. To assess their performance in sodium‐ion batteries, Li et al. [287] used *Eucalyptus* hardwood, scots pine softwood, and two other biomasses, moso bamboo and juncao, to produce ACs. Bamboo‐derived ACs were found to be the most suitable anode material for use in sodium‐ion batteries with respect to the other precursors used. The rate performance, electrochemical impedance spectroscopy (EIS) measurements, and cycling performance of the ACs prepared using different types of biomasses are shown in Figure 10. The high performance shown by bamboo‐derived carbons was associated with the presence of carbonyl groups on its surface as well as its highly disordered pseudo‐graphitic nature. At a current density of 20 mA g^−1^, the reversible capacity reached 344.3 mAh g^−1^, and after 100 cycles, the stability remained at 82.6% at 1 A g^−1^.

The power density of cherry wood‐based ACs used in AABs is ~267 mW cm^−2^ higher than that of carbon fiber paper‐based electrodes (~236 mW cm^−2^) [29]. Comparisons of the performance of batteries made with ACs prepared using different kinds of wood and other biomasses are listed in Table 5.

**TABLE 5:** Comparison of the properties obtained by different kinds of batteries developed using different types of biomasses.

Type of precursor	Used biomass	As a component used in battery type	Used electrolyte	Best reported results of battery performance		References
				Power density	Energy density/reversible capacity	
*Hardwood*	Balsa (*Ochroma*)	Current collector as well as a cathode for LABs	1 M LiTFSI dissolved in TEGDME	—	3316 mA h g^−1^ at 200 mA g^−1^	[30]
	Cherry (*Prunus avium*)	Current collector for AABs	0.1 M KOH	267 mWcm^−2^ at 15 mA cm^−2^	—	[29]
	*Eucalyptus*	Anode for SIBs	1 M NaPF_6_/DEGDME	—	303.5 mAh g^−1^ at 20 mA g^−1^	[287]
	Kraft lignin (commercially available)	Anode for SIBs	1 M NaClO_4_ solution in EC + PC with fluoroethylene carbonate	—	231.7 mAh g^−1^ at 30 mA g^−1^	[288]
	Birch	Anode for LIBs	1 M LiPF_6_ in EC/DMC (1:1 by volume)	—	319 mAh g^−1^ at 1C	[289]
		Anode for SIBs	1 M NaPF_6_ in EC/PC	—	233 mAh g^−1^ at 100 mA g^−1^	
	Locust	Anode for SIBs	1 M NaClO_4_ in EC and DMC solution	—	382.3 mAh g^**−**1^ at 0.3C	[290]
*Softwood*	Pine wood (*Pinus sylvestris*)	Cathode for AABs	1 M KOH and 1 M NaCl	230 mWcm^−3^	640.0 W h kg^−1^ at discharge rate of 200 mA cm^−3^	[31]
	Radiata pine tree (*Pinus radiata*)	Anode for ZIBs	2 M ZnSO_4_	—	—	[32]
	Scots pine (*Pinus sylvestris*)	Anode for SIBs	1 M NaPF_6_/DEGDME	—	309.2 mAh g^−1^ at 20 mA g^−1^	[287]
	Pine wood (*Pinus sylvestris*)	Anode for SIBs	1 M NaPF_6_ in EC, PC, DMC solution		372 mAh g^−1^ at 18.6 mA g^−1^	[291]
	Pine wood (*Pinus sylvestris*)	Anode for SIBs	1.0 M NaPF6 in DEGDME		314 mAh g^−1^ at 1C	[292]
*Other biomass*	Rice husk	Cathode for AABs	NaCl	—	—	[33]
	Peanut shell	Air cathode for AABs	KOH	1.78 Wm^−2^	787.0 kJ g^−1^ at 1mA g^−1^	[282]
	Chestnut shell	Cathode for lithium‐sulfur batteries	1 M LiTFSI with LiNO_3_ as additive	—	1537.7 mAh g^−1^	[293]
	Bamboo (*Banbusa*)	Anode for SIBs	1 M NaPF_6_/DEGDME	—	344.3 mAh g^−1^ at 20 mA g^−1^	[287]
	Juncao straw	Anode for SIBs	1 M NaPF_6_/DEGDME	—	273.2 mAh g^−1^ at 20 mA g^−1^	
	Olive shell	Anode for Sodium ion fuel cell	1.0 M NaClO_4_ in EC and DEC solution	—	320 mAh g^−1^ at 50mAg^−1^	[252]
	Sheaths of *Areca catechu*	Anode for SIBs	NaClO_4_ in EC and DEC solution	—	676 mAh g^−1^ at 0.134 C	[294]

Lithium bis(trifluoromethanesulfonyl)imide (LiTFSI).

Tetraethylene glycol dimethyl ether (TEGDME).

Sodium hexafluorophosphate (NaPF_6_).

Sodium perchlorate (NaClO_4_).

Lithium hexafluorophosphate (LiPF_6_).

Lithium bis (trifluoromethane sulfonyl) imide (LiTFSI).

Lithium nitrate (LiNO_3_).

Ethylene Carbonate (EC), Propylene Carbonate (PC).

Dimethyl Carbonate (DMC), Diethylene Glycol Dimethyl Ether (DEGDME).

Diethyl Carbonate (DEC).

ACs derived using hardwood, softwood, and other biomasses have been used as different components (anode or cathode) in various types of batteries. They provide excellent performance in terms of sufficient power density and high energy. ACs are used as electrode materials in supercapacitors, as described in the next section.

### 5.2 ACs in Supercapacitors

Electrode materials derived from ACs, transition metal oxides, and conductive polymers are widely used in different types of supercapacitors (symmetric, asymmetric, and hybrid) [23, 24, 25, 26, 27, 28]. However, ACs derived from the activation process of wood show favorable electrochemical properties when used as electrode materials in supercapacitors because of the presence of many lattice defects. Porous and nonporous electrode materials have long been tested in supercapacitor applications. Luo et al. [263] synthesized heteroatom‐doped hierarchical porous carbons using hardwood (*Quercus variabilis*) biomass and used them as electrode materials in supercapacitor applications. Using a 1:1 ratio of H_3_PO_4_ as an impregnating agent to the biomass precursor, the surface area of heteroatom‐doped synthesized ACs was reported to be 515.80 m^2^ g^−1^, with a pore size of 1.4 nm. Heteroatoms present on the carbon surface generated polar sites, which improved the capacitive performance by facilitating good wettability of micropores by electrolyte ions and reducing the impedance at the electrolyte–electrode interface [132, 263]. A supercapacitor prepared via this optimized AC with 6 M KOH could deliver 206 F g^−1^ of specific capacitance at 0.5 A g^−1^ current density and 15.05 Wh kg^−1^ of energy density at a power density of 563 W kg^−1^ [263]. Ayinla et al. [99] prepared AC electrodes using softwood pine bark (*Pinus sylvestris*) as a precursor via different impregnating agents, such as H_3_PO_4_, potassium carbonate, and ZnCl_2_. Compared with other agents, H_3_PO_4_ was reported to be the best impregnating agent because a supercapacitor designed using H_3_PO_4_ impregnated electrodes and a 6 M KOH electrolyte gave a maximum specific capacitance of 578.5 F g^−1^ in a three‐electrode system. Later, these electrodes were evaluated in a two‐electrode system with two different electrolytes, namely, 6 M KOH and Cu^2+^ cellulose nanofiber hydrogels. These electrodes were best fit with Cu^2+^ cellulose nanofiber hydrogel electrolytes, with a specific capacitance of 157.2 F g^−1^ at a current density of 0.5 A g^−1^ and a power density of 1156.4 W kg^−1^ at an energy density of 14.75 Wh kg^−1^ [99]. The ACs derived from different kinds of biomasses used in supercapacitor applications reported by various research groups are listed in Table 6.

**TABLE 6:** Comparison of the properties obtained by supercapacitors developed using different types of biomasses.

Type of precursor	Biomass	Electrolyte	Best reported results of supercapacitor performance				References
			Charge transfer resistance (Ω)	Maximum specific capacitance (F g^−1^)	Power density (W kg^−1^)	Energy density (Wh kg^−1^)	
*Hardwood*	*Quercus variabilis*	6 M KOH	0.52	206 at 0.5 A g^−1^	563	15.03	[263]
	Jackwood (*Cryptocarya glaucescens*) (Medium hardwood)	1 M H_2_SO_4_	5.31	147.2 at 0.5 mA cm^−2^	68.5	8.02	[22]
	Birchwood (*Betula*)	H_2_SO_4_	—	308	—	—	[3]
	*Terminalia tomenalia* (*MnO*_2_ *modified*)	6 M KOH	—	484.3 at 1 A g^−1^	150.4	24.2	[295]
	*Eucalyptus*	2 M NaCl	0.19	230 at 1 A g^−1^	2396	41	[258]
Softwood	Pine (*Pinus sylvestris*) bark	Cu^2+^‐cellulose nanofibers hydrogel	—	157.2 at 0.5 A g^−1^	1156.4	14.75	[99]
	*Cedrus deodara* (*MnO*_2_ *modified*)	6 M KOH	—	309.2 at 1 A g^−1^	148.4	16.8	[295]
Other biomass	Sterculia foetida's fruit shell	1 M H_2_SO_4_	0.5	308.6 at 1 A g^−1^	1200	27.66	[101]
	Camphor tree grains	6 M KOH	—	423 at 1 A g^−1^	250	12.5	[143]
	Pomelo peel	6 M KOH	0.65	247.26 at 0.5 A g^−1^	—	—	[102]
	Corn straw	1 M Na_2_SO_4_	—	227 at 0.2 A g^−1^	—	20.2	[141]
		6 M KOH	—	327 at 100 A g^−1^	—	—	
	Peanut shell	EMITf mixed PVDF‐HFP and Mg(Tf)_2_/EMITf mixed PVDF‐HFP	29	189 at 1 mA cm^−2^	57	26	[111]
	Melon peels	PVDF‐HFP/1 M LiTf in EMITF	9	170 at 1 A g^−1^	860	31.34	[112]

### 5.3 ACs in Fuel Cells (FCs)

Microbial fuel cells (MFCs) are based on microbial metabolism. The operation of an MFC is based on converting organic waste into electricity. The microbial metabolism process used in MFCs, where organic matter breaks down with the release of an electron as a byproduct and generates electricity. Conventional membranes used in MFCs have high internal resistance, and conventional anodes suffer from low surface area, which affects the power density of the MFCs; therefore, the use of MFC technology requires the development of sustainable, low‐cost, and high‐performance materials in which wood‐derived anodes play an important role [296]. A membrane‐free MFC with wood‐derived (grape vine branches) carbon as the anode has been designed to avoid the formation of biofilms, which generally develop on the cathode side under the action of aerobic microorganisms and stop the functioning of the cell. These grape vine branch‐based carbons used as anodes in membrane‐free MFCs generated a power of nearly 510 mW m^−2^ when sewage‐driven air cathodes were used [296]. In another study, an MFC was realized using a cathode of sycamore (*Platanus orientalis*)‐derived carbon with a power density of 1500 mW m^−2^ [40]. In this study, the performance of wood‐based cathodes was reported to be nine times better than that of conventional cathodes, such as carbon felt, with a maximum power density of 1500 mW m^−2^ achieved. Basswood‐derived carbon electrodes have been efficiently used in MFCs to treat waste‐water [297] and are better than other commercial carbon cloths or carbon felts. After 48 h of treatment, all the chromium was removed from 20 mg L^−1^ concentrated Cr(VI) wastewater.

Solid oxide fuel cell (SOFC) technology is being improved with direct carbon solid oxide fuel cells (DC‐SOFCs) using AC directly as fuel [298, 299]. Red shell biomass and its biochar have been used as fuel and biochar‐fueled cells and have shown better performance, with an OCV of 1.08 V and a peak power density of 719 mW cm^−2^, than biomass‐fueled cells, with an OCV of 1.03 V and a peak power density of 619 mW cm^−2^ [298]. Compared with electrolyte‐supported FCs, the use of either biomass or biochar as fuel in DC‐SOFCs results in better performance of the cell. The presence of sufficient K and Ca in red shell biomass makes it a naturally existing metal catalyst, and its woody nature results in a disordered structure with numerous pores, making it a suitable candidate for DC‐SOFCs. *Eucalyptus* char is used as a fuel in SOFCs and delivers a power density of 826 mW cm^−2^ [299].

**FIGURE 11:**
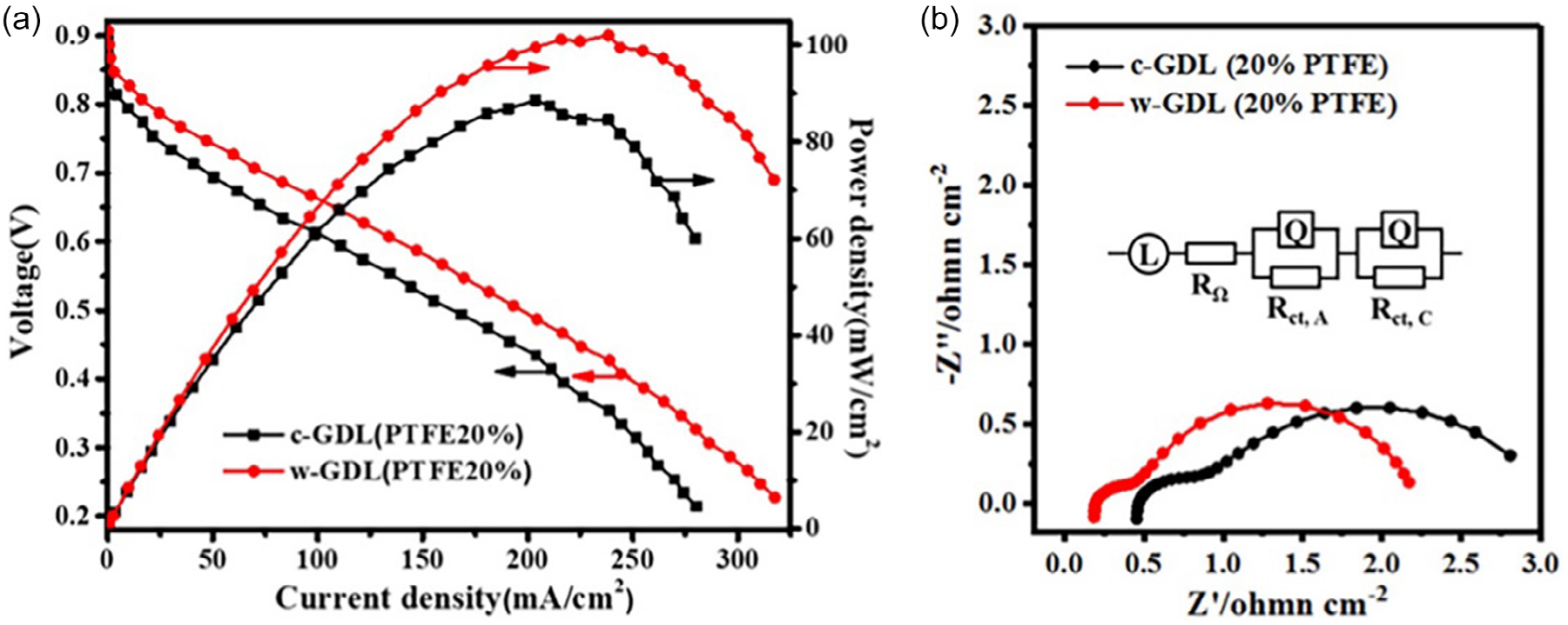
Air‐breathing PEMFC assembled using wood‐derived GDL (w‐GDL) and carbon paper‐derived GDL: (a) the variation of potential and power density with current density, and (b) EIS spectra of fuel cells designed using pine wood‐based and carbon paper‐based GDLs. Reproduced from ref. [303]. Copyright (2021), Elsevier B.V.

Proton exchange membrane fuel cells (PEMFCs) are known for reducing carbon emission when they are used in various applications, particularly for generating clean energy using hydrogen as fuel [300]. Effective electrocatalysts and suitable support materials are required for PEMFCs. For a long time, platinum has been the best electrocatalyst material, but it is costly. Carbon black is a known support material but it suffers from corrosion if it is used with platinum, and weakens carbon and platinum particle bonding. Considering such problems, wood‐based carbon/biochar has been proven to be a good support material [300]. The GDL is one of the crucial components of air‐breathing PEMFCs. Open cathodes through the GDL should be able to effectively conduct gas convection and diffusion for the proper functioning of reactions. In terms of function, water is required to hydrate the proton‐conducting membrane; however, excess water can block electrode pores and restrict gas diffusion [301]. Carbon cloth or carbon fiber paper with PTFE as a binder is being used as a conventional GDL but has indicated difficulties with mass transfer and water management because it has disordered pores and is responsible for voltage drop and concentration polarization [302]. Natural WDACs can be suitable candidates for use as GDLs, as their ordered low τ channels can shorten gas diffusion pathways and resolve water clogging problems because of their aligned tube‐like structure. The diffusion kinetics in wood‐derived GDLs become easier than those in conventional GDLs. Pine block wood‐based GDLs have been used in PEMFCs and reported to be more efficient than other conventional GDLs based on carbon cloth [303]. A PEMFC derived using a pine wood‐derived GDL delivered a current density of 0.139 A cm^−2^ at 0.6 V with a maximum power density of ~0.102 W cm^−2^ at 0.43 V. A comparison of the polarization and power density curves and impedance curves for the air‐breathing PEM fuel cell prepared using a pine wood‐based GDL and carbon paper‐based GDL is presented in Figure 11. The generated power density of fuel cells based on various kinds of biomasses used, compared with conventionally used electrodes such as carbon cloth and carbon felt, is given in Table 7.

**TABLE 7:** Comparison of materials used as components in fuel cells and their performance when different types of biomasses are used.

Wood category	Fuel cell type	Cathode	Anode	Power density (mW m^−2^)	References
Hardwood	MFC	Sycamore wood (*Platanus orientates*)	Carbon felt	1500.00	[40]
		Bass wood (*Tilia americana*)	Carbon felt	62.59	[297]
		Balsawood (*Ochroma*)	Carbon felt	200.00	[304]
		Carbon felt	Basswood (*Tilia americana*)	3040.00	[305]
Softwood		CNTs/AC	Grape wood (*Vitis vinifera* L.)	516.00	[296]
		CNTs/AC	Carbon paper	100	
		CNTs/AC	Carbon cloth	135	
		CNTs/AC	Carbon felt	142	
		Carbon cloth	Pine wood (*Pinus*)	428.60	[306]
		Carbon felt	Cedar wood (*Cedrus*)	9.90	[307]
Biomass	Alkaline direct methanol fuel cell	Almond shell (Fe‐N modified)	PtRu/C	21.00	[308]
	MFC	Bauhinia accuminata AC coated stainless steel mesh	Mango seed hull modified carbon veil	3.34	[309]

### 5.4 ACs for Other Applications

Dye removal remediation is an important issue need that must be addressed by different industries, such as the dyestuff, rubber, textile, cosmetics, plastics, and paper industries, as well as in solving environmental problems related to cleaning contaminated water. Cationic and anionic dyes are not safe for aquatic environments. Dye adsorption processes require materials called adsorbent materials, which can easily bind dye molecules through chemical, physical, or ionic binding [310]. Few agricultural waste materials have been directly used in dye removal to avoid the use of any chemical use and to make the process cost‐effective. Peel from grapefruit and yellow passion fruit has been successfully used as a raw dye absorbent from aqueous solutions without any modification [311, 312]. Moreover, biochar derived from wood with higher density may have higher porosity and therefore might be more useful for the sorption of heavy metals [313].

ACs representing more micropores with the presence of mainly oxygen functional groups are more useful for adsorption studies [314]. Hardwood‐derived AC with respect to softwood derived once may present different porosities and specific surface areas. Ge et al. [65] compared the catalytic reduction performance of hardwood‐ and softwood‐derived ACs for industrial 4‐nitrophenol. They developed Pd‐decorated ACs using *Pinus sylvestris* as a softwood precursor and basswood as a hardwood precursor and reported better catalytic solubility for hardwood‐derived ACs than for softwood‐derived ACs. The mass transfer efficiency depends on the ratio of mesopores to micropores. They reported that more mass transfer was possible because of the abundant interpenetrating channels present in hardwood, and resulted in better catalytic reduction [65]. Functional groups such as carboxyl, hydroxyl, methoxy, and phenol groups are useful for binding dye molecules; however, other parameters, such as the pH and temperature of the solution, must be considered [43].

Apart from all scientific and technological advancement of ACs discussed above, the environmental and economic aspect of carbons is also an important area of interest. Therefore, the life cycle assessment of ACs is discussed in next section.

### 5.5 Life Cycle Assessment of ACs

Recent life‐cycle assessment (LCAs) highlights that ACs derived from biomass can substantially reduce environmental burdens compared with conventional coal‐based products. Gu et al. [315] reported that AC from woody residues produced via pyrolysis and steam activation exhibited approximately 35% lower cumulative energy demand and more than 50% lower greenhouse gas (GHG) emissions than coal‐based AC, mainly due to biogenic carbon and reduced fossil energy use. Similarly, Arena et al. [59] demonstrated that AC production from coconut shells in Indonesia offers notable advantages in terms of global warming and acidification potentials, particularly when renewable electricity is employed. Their sensitivity analysis showed that substituting fossil‐derived power with biomass‐based electricity can cut GHG emissions by up to 80%. Together, these studies confirm that utilizing lignocellulosic residues as AC precursors enhances carbon efficiency and supports circular bioeconomy strategies, although energy sourcing remains a key determinant of overall sustainability.

## 6 Conclusion

The synthesis of ACs/biochars via different types of waste materials has remained an important topic among researchers for many decades. However, the type of biomass and the adopted synthesis process of carbons can notably affect the final properties of the produced ACs. WDACs are becoming more important because of the high availability of wood waste. Woods are biocompatible, biologically safe, environmentally friendly, and sustainable resources. In this review, the effects of different steps and parameters affecting the properties of ACs are described in detail. We have categorized wood types and collected information on the properties of ACs derived using softwood and hardwoods along with other PBBs. However, many studies have been performed on various biomasses and woods using different synthesis processes in terms of the use of impregnating agents, activation temperatures, and pre‐ and post‐activation processes. However, these studies are random in terms of the activating agents and other controlling parameters. Therefore, at this point, there is a limitation as it is still difficult to understand how to choose a particular species for a certain application. The application of ACs in different types of energy devices is also described. Modern technology requires higher energy density and long cycle life batteries where biochar or pure ACs may limit their performance. ACs modification via tuning with heteroatom doping or surface treatments is compulsory to enhance their electrical properties to make them suitable materials for high current applications. This may enhance synthesis cost as well as environmental impact too. High temperature treatments (>1000°C) help to improve degree of graphitization but they have other challenges like carbon pore collapse, improper doping and also increased energy cost. Another challenge is low yield after pyrolysis which restricts ACs use at large scale. Effective pre‐treatments are being tried to enhance its yield but still require more efforts. Further research is required towards optimizing the synthesis process to obtain AC with certain properties and large‐scale production.

## Author Contributions

**Meenal Gupta**: writing – original draft and editing, writing – review and editing, visualization, conceptualization. **Maria F. Gaele**: writing – review and editing. **Pasquale Gargiulo**: writing – review and editing. **Yogesh Kumar**: writing – review and editing, supervision. **Valeria Califano**: writing – review and editing. **Aniello Costantini**: writing – review and editing. **Tonia M. Di Palma**: writing – review and editing, supervision, conceptualization, project administration, funding acquisition.

## Funding

This study was supported by Ministero dell'Università e della Ricerca (P20227BLHS and PNC0000007) and University of Delhi (PRABODH scheme grant no. 140).

## Conflicts of Interest

The authors declare no conflicts of interest.
